# Schistosoma-Salmonella co-infection in Africa: Epidemiology, biological mechanisms, and antimicrobial resistance: A systematic review and meta analysis

**DOI:** 10.1371/journal.pone.0359975

**Published:** 2026-10-29

**Authors:** Brhane Berhe, Hadush Negash, Tesfay Abadi, Getachew Belay, Merhawi Alemu, Aberham Abere, Haftom Legese, Abel Deres

**Affiliations:** 1 Department of Medical Laboratory Science, College of Medicine and Health Sciences, Adigrat, Ethiopia; 2 Department of Medical Parasitology, School of Biomedical and Laboratory Sciences, College of Medicine and Health Sciences, University of Gondar, Gondar, Ethiopia; Mekdela Amba University, ETHIOPIA

## Abstract

Schistosomiasis and salmonellosis are major public health problems in Africa, contribute substantially to morbidity. Schistosoma-Salmonella co-infection is a neglected and overlooked health condition with important clinical and public health implications. Therefore, we conducted a systematic review and meta-analysis to synthesize evidence on the epidemiology, biological mechanisms, and antimicrobial resistance of Schistosoma-Salmonella co-infection in Africa. A systematic review and Meta-analysis were conducted following the PRISMA framework. The pooled prevalence of co-infection was estimated using a random effects model, with at 95% confidence intervals. Between studies heterogeneity was assessed using Cochran’s Q test and the I^2^ statistic. Subgroup analyses were performed according to Schistosoma species and country to explore potential source of heterogeneity. This systematic review found that the pooled prevalence of Schistosoma-Salmonella co-infection in Africa was 9%. Species specific pooled prevalence was 4% for *Schistosoma mansoni*-Salmonella co-infection and 12% for *Schistosoma hematobium*-Salmonella co-infection. Country based subgroup analysis showed prevalence of *Schistosoma mansoni*-Salmonella co-infection of 2% in Ethiopia, 9% in Sudan, 2% in Democratic republic of Congo and 4% in Nigeria, while *Schistosoma hematobium*-Salmonella co-infection prevalence were 0%, 25%, and 8% in Ethiopia, Sudan, and Nigeria respectively. Schistosoma-Salmonella co-infection is driven by immune modulation, tissue pathology, and parasite bacterium interactions that support persistent infection and compromise therapeutic efficacy, thereby contributing to treatment failure and the emergence of antimicrobial resistance. Schistosoma-Salmonella co-infection represents an important and under-recognized public health concern in Africa, with a pooled prevalence of 9%. The findings demonstrate notable variation in co-infection prevalence across Schistosoma species and geographical settings, with higher rates observed among *Schistosoma haematobium* compared with *Schistosoma mansoni*. Strengthened surveillance systems, improving early diagnosis, and implementing integrated control strategies are essential to reduce the burden and enhance clinical management of this neglected co-infection in Africa.

## 1. Background and justification

Schistosomiasis is a neglected tropical disease caused by blood flukes, affecting over 250 million people worldwide, with about 90% of cases occur in Africa [1]. The disease is mainly due to *Schistosoma mansoni* and *Schistosoma haematobium* in Africa [1,2].

Salmonellosis is another important infectious disease causing significant morbidity and mortality in Africa and remains a major cause of bloodstream and gastrointestinal infections particularly in children [3]. The burden is aggravated by delayed diagnosis, poor laboratory infrastructure, and increasing antimicrobial resistance [4].

Schistosomiasis and salmonellosis coexist in many African countries where both diseases remain endemic, yet Schistosoma-Salmonella co-infection remains poorly understood [1,3]. Geographic distribution, variation in control program, inadequate sanitation, and unsafe water sources are the contributing factors for persistent of the co-infection [1,2]. However, reports showed variation in prevalence across African countries [5,6].

The biological interaction mechanisms underlying Schistosoma-Salmonella co-infection remain a major challenge. Previous studies have demonstrated that Salmonella organisms can attach to adult Schistosoma worms, enhancing bacterial survival by facilitating immune evasion and reducing susceptibility to antimicrobial therapy [5]. This interaction may contribute to prolonged infection and increased disease burden, particularly in endemic regions of Africa [6]. However, in Schistosoma- endemic areas, co-infections are likely under-reported due to limited diagnostic capacity, insufficient clinical awareness, and overlapping clinical manifestations.

Antimicrobial resistance of Salmonella is an emerging public health concern in Africa and poses additional challenges for effective treatment [4]. The presence of Schistosoma-Salmonella co-infection may further compromise therapeutic outcomes by promoting bacterial persistence, immune evasion and recurrent infection [5]. However, comprehensive evidence regarding the epidemiology, biological mechanisms, and antimicrobial resistance of Schistosoma-Salmonella co-infection remains limited. This knowledge gap hinders clinical management, public health surveillance, and the development of effective control strategies. Therefore, we aimed to conduct a systematic review and meta-analysis of the epidemiology of Schistosoma-Salmonella co-infection in Africa, and synthesize evidence on its biological mechanisms, and antimicrobial resistance implications.

## 2. Materials and methods

### 2.1. Study setting

This systematic review was conducted to study the epidemiology, biological mechanisms, and antimicrobial resistance of Schistosoma-Salmonella co-infection in Africa. It is the world largest continent by geographic area and the second most populous continent in the world next to Asia, covering approximately 30.37 million km^2^ and hosting more than 1.56 billion people [7].

### 2.2. Protocol design

This systematic review summarized evidence exclusively from observational studies with a cross-sectional study design conducted across different regions of Africa. It was performed in accordance with the Preferred Reporting Items for Systematic Reviews and Meta-analyses (PRISMA) guidelines (Fig 1) and PRISMA checklist (S1 File) [8] and meta-analysis of observational studies in epidemiology guidelines [9]. PRISMA was used to guide the transparent reporting of the identification, screening, eligibility assessment, and inclusions of the studies. The study selection process was presented using a PRISMA flow diagram, which documented the number of records identified, screened, excluded, and included in the final review. Despite the PRISMA checklist was used, a formal review protocol was not registered.

**Fig 1:**
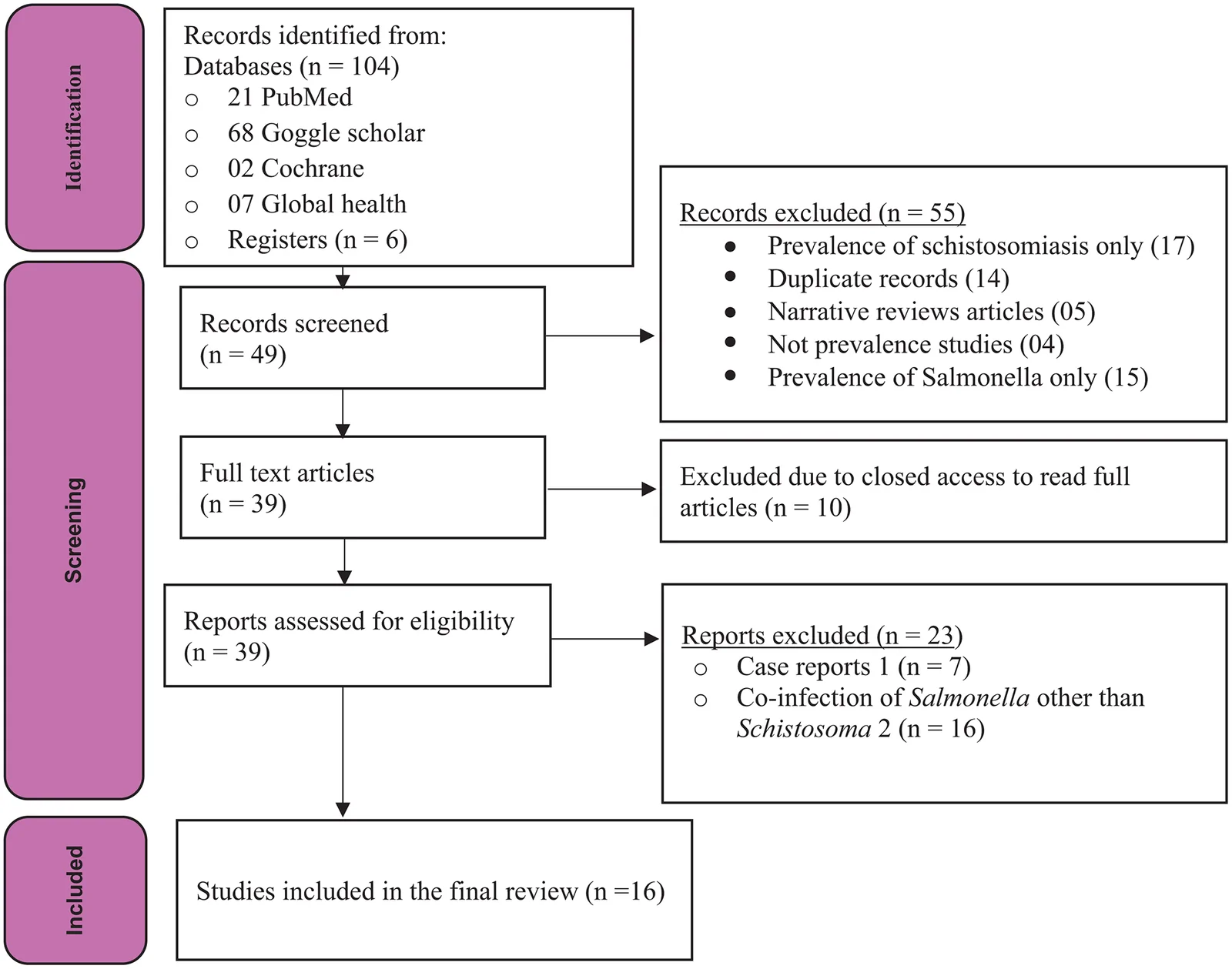
PRISMA follow diagram showing the review selection procedures of articles for the Epidemiology of Schistosoma Salmonella co-infection in Africa, 2026.

### 2.3. Sources and search strategies

A comprehensive literature search was conducted in PubMed, Google Scholar, EMBASE, Cochrane, Global Health and CINAHL to identify studies reporting on Schistosoma-Salmonella co-infection in Africa. The searches were performed from database inception to July 5, 2026, without date restrictions. Only studies published in English were considered. The search strategy was developed using controlled vocabulary (e.g., MeSH terms) and free text terms related to Schistosoma, Salmonella, co-infection and Africa. Synonyms, spelling variants, truncation, and wildcard operators were incorporated where supported by the respective databases. Boolean operators “AND” and “OR” were used to combine search concepts. The search strategy was adapted to the syntax and controlled vocabulary of each database and independently reviewed by three authors.

We used the following search words in PubMed: (“schistosomiasis”[MeSH Terms] OR Schistosomiasis[Text Word] OR “schistosoma”[MeSH Terms] OR Schistosoma[Text Word]) AND (“salmonella”[MeSH Terms] OR Salmonella[Text Word]) AND (“coinfection”[MeSH Terms] OR co-infection[Text Word]) AND (“africa”[MeSH Terms] OR Africa[Text Word]) AND (“epidemiology”[Subheading] OR “prevalence”[MeSH Terms] OR Prevalence[Text Word]) AND (“drug resistance, microbial”[MeSH Terms] OR “antibiotic resistance”[Text Word]) AND (Biological [Text Word] AND mechanisms[All Fields]). We also used keywords such as “Prevalence and *Schistosoma mansoni* co-infection”, “Epidemiology and *Schistosoma mansoni*-Salmonella co-infection”, “Incidence and *Schistosoma mansoni*-Salmonella co-infection”, “Prevalence and *Schistosoma haematobium*-Salmonella co-infection”, “Epidemiology and *Schistosoma haematobium*-Salmonella co-infection”, and “Incidence and Urinary schistosomiasis Salmonella co-infection in Africa”.

To minimize the risk of missing potentially relevant studies and to identify unpublished data, supplementary searches were also conducted. Reference lists of all included studies and relevant systematic and narrative reviews were manually screened for additional eligible studies. Google Scholar was searched using combinations of terms related to *Schistosoma*, *Salmonella*, co-infection, and Africa. Relevant websites of international and national organizations, including the World Health Organization (WHO), the Centers for Disease Control and Prevention (CDC), and Ministry of Health (MOH), were screened for potentially eligible reports. In addition, the Conference Proceedings Citation Index-Science (CPCI-S) was searched for relevant proceedings of the International Union Against Schistosomiasis conferences, and ProQuest Dissertations & Theses was also searched for relevant dissertations and theses. These supplementary searches were performed to identify additional published, unpublished, and grey-literature sources that may not have been retrieved through the electronic database searches.

### 2.4. Study selection and data extraction

After the entire literature search through the database and supplementary searches, all the Literatures were imported to EndNote X7.8 (Bid 7705) for reference management and duplicate removal. The collected data were purposefully processed via Microsoft Excel 2007. Two researchers independently screened studies for eligibility according to the predefined eligibility criteria. The full texts of potentially eligible studies were subsequently retrieved and independently assessed by the same two reviewers. Following screening, studies were selected irrespective of their design and time if they met the inclusion criteria. The researchers autonomously reviewed titles and abstracts against eligibility criteria to categorize the article as either ‘to include or ‘exclude. A third researcher was consulted for discrepancies at each stage of the selection. We resolved differences in opinion through discussion.

Data were extracted two researchers from each selected study via a predetermined list of categories, including first author, year of publication, Country, setting (community/health facility), study design, prevalence of Schistosoma-Salmonella co-infection, prevalence of *Schistosoma mansoni*-Salmonella co-infection, prevalence of *Schistosoma hematobium*-Salmonella co-infection, type of specimen used, and laboratory method used, biological mechanism of Schistosoma-Salmonella co-infection reported, antimicrobial resistance on Schistosoma-Salmonella reported via Microsoft Excel 2007.

### 2.5. Handling of missing data

We requested corresponding authors through electronic mail and ResearchGate to provide missing outcome data for some studies.

### 2.6. Eligibility criteria

For the systematic review, articles with human subjects who were available through open access, assessed for both Schistosoma and Salmonella infection, conducted in Africa, reported at least one outcome relevant to the review, including the prevalence or epidemiological characteristics of Schistosoma-Salmonella co-infection, species-specific co-infection involving *Schistosoma mansoni* or *Schistosoma haematobium*, briefly demonstrating study selection and data extraction, had a quality assessment score of 50%, and were sufficiently relevant to satisfy the review selection criteria were included for the Meta analysis where as for the biological mechanisms and antimicrobial resistance pattern, human and animals subject were used to narrate the mechanisms. Only studies published in English were included. No restriction was applied to the year of publication.

### 2.7. Quality assessment and critical appraisal

The quality of each study included in the review was assessed via a critical appraisal tool adopted from the Joanna Briggs Institute (JBI) checklist of 2017 to assess the validity and quality of studies, the recommended tool for evaluating primary studies for inclusion in systematic reviews for etiology and risk review [10]. A third researcher was consulted to resolve discrepancies while appraising the quality assessment. The items used for the quality assessments are presented in (S2 File).

### 2.8. Statistical data analysis

R statistical software version 4.5.1 (R Foundation for Statistical Computing, Vienna, Austria) was utilized to carry out the Meta-analysis. A Meta-analysis of proportions was conducted to estimate the pooled prevalence of Schistosoma-Salmonella co-infection with corresponding 95%-CI. Considering the expected clinical and methodological heterogeneity across studies, a random-effects model was applied. Between-study variance (τ^2^) was estimated using the DerSimonian-Laird method. To alleviate the variance of proportions, a variance-stabilizing transformation was applied prior to pooling. Heterogeneity among studies was assessed using Cochran’s Q test and quantified using the I^2^ statistic. An I^2^ value greater than 50% was considered indicative of substantial heterogeneity. A two-sided P-value <=0.05 was considered statistically significant.

**Ethical statement** Not applicable.

### 2.9. Sub-group analysis

Subgroup analyses were conducted to explore potential sources of heterogeneity by study countries. Sensitivity analyses were also performed to assess the robustness of the pooled estimates by sequentially excluding each individual study from the meta-analysis. Publication bias was not assessed because the number of included studies was fewer than the minimum of 10 studies generally recommended for reliable assessment of publication bias.

### 2.10. Assessment of heterogeneity, and publication bias

Statistical heterogeneity among the included studies was assessed using forest plots with 95% prediction with chi-square (χ^2^), P value and I-square (I2) tests [11]. I^2^ values were interpreted as measures of the proportion of total variability attributable to between-study heterogeneity. Publication bias were not formally assessed using statistical tests of funnel-plot asymmetry because fewer than 10 studies were available for the relevant meta-analyses [12]. This decision was based on the limited statistical power and reliability of funnel-plot asymmetry tests when the number of studies is small. Therefore, no formal Egger’s test was performed. Funnel plots were not used as a formal test of publication bias.

## 3. Results

### 3.1. Overview of the systematic and Meta analysis review

This systematic review identified 104 publication records through a database search. Among these records, 17 research articles on the prevalence of Schistosoma-Salmonella co-infections, 56 articles on the prevalence of *Schistosoma mansoni*-Salmonella co-infections, and 31 articles were on the prevalence of *Schistosoma hematobium*-Salmonella co-infections. After duplicates were removed, 11 studies on Schistosoma-Salmonella co-infections, 23 articles on *Schistosoma mansoni*-Salmonella co-infections and 15 studies articles on *Schistosoma hematobium*-Salmonella co-infections were screened on the basis of their titles and abstracts. After screening 10 articles were excluded due to closed access to read full text articles. Nine articles for the prevalence of Schistosoma-Salmonella co-infections, 20 articles for *Schistosoma mansoni*-Salmonella co-infections and 10 articles for *Schistosoma hematobium*-Salmonella co-infections were assessed as eligible for full-text reviews. After a comprehensive reading, 7 articles on the prevalence of Schistosoma-Salmonella co-infections, 10 articles on the prevalence of Schistosoma mansoni-Salmonella co-infections and 9 studies on the prevalence of *Schistosoma haematobium*-Salmonella co-infections met the inclusion criteria and were included in this systemic review and meta-analysis. The list of studies included is provided in Tables 1–Table 3. The review selection process was illustrated via a PRISMA diagram (Fig 1).

**Table 1:** Results on the prevalence of Schistosoma-salmonella co-infections in Africa.

Author	YP	Country	SD	SZ	P	SU	LM	QS (%)
Obi AA et al.[13],	1996	Nigeria	CS	268	45	Urine, Stool and Blood	Microscope+Widal	100
Aemero M et al., [14]	2012	Ethiopia	CS	634	0	Urine and Stool	Microscope+culture	100
Beyene G et al., [15]	2014	Ethiopia	CS	260	0	Stool	Microscope+culture	100
Lam PM et al., [16]	2006	Nigeria	CS	410	24	Urine and Stool	Microscope+culture	100
Mohager MO et al., [17]	2014	Sudan	CS	288	64	Urine and Stool	Microscope+Culture	100
Salem AK et al., [6]	2015	Sudan	CS	203	30	Urine, Stool and Blood	Microscope+culture	100
Yandev et al., [18]	2022	Nigeria	CS	180	8	Urine and Stool	Microscope+Culture	100

YP-Year of publication, SD-Study design, CS- Cross sectional study, SZ- Sample size, P-Total positive, SU-Specimen used, LM-Laboratory method used, QS-Quality appraisal.

**Table 2:** Results on the prevalence of *Schistosoma mansoni*-Salmonella co-infections in Africa.

Author	YP	Country	SD	SZ	P	SU	LM	QS (%)
Aemero M et al., [18]	2012	Ethiopia	CS	634	0	Urine and Stool	Microscope+Widal	100
Beyene G et al., [15]	2014	Ethiopia	CS	260	0	Stool	Microscope+Culture	100
Elfaki TEM et al., [19]	2015	Sudan	CS	75	17	Stool and Blood	Microscope+Culture	100
Kalonji LM et al., [20]	2020	Congo	CS	1108	24	Stool and Blood	Microscope+culture	100
Lam PM et al., [16]	2006	Nigeria	CS	410	18	Urine and Stool	Microscope+Culture	100
Marege M et al., [21]	2021	Ethiopia	CS	271	21	Stool and Blood	Microscope+Culture	100
Modebe AA et al. [22]	2014	Nigeria	CS	250	6	Stool and Blood	Microscope+Widal+Culture	100
Mohager MO et al., [17]	2014	Sudan	CS	288	6	Urine and Stool	Microscope+Culture	100
Salem AK et al., [6]	2015	Sudan	CS	203	7	Urine, Stool and Blood	Microscope+Culture	100
Igwe NN et al., [23]	2014	Nigeria	CS	500	27	Urine, Stool and Blood	Microscope+ICT	100

YP-Year of publication, SD-Study design, CS- Cross sectional study, SZ- Sample size, P-Total positive, SU-Specimen used, LM-Laboratory method used, QS-Quality appraisal.

**Table 3:** Results on the prevalence of *Schistosoma hematobium*-Salmonella co-infections in Africa.

Author	YP	Country	SD	SZ	P	SU	LM	QS (%)
Abubakar IM et al., [24]	2025	Nigeria	CS	410	4	Urine and Stool	Microscope+Culture	100
Aemero M et al., [14]	2012	Ethiopia	CS	634	0	Urine and Stool	Microscope+Culture	100
Beyene G et al., [15]	2014	Ethiopia	CS	260	0	Stool	Microscope+Culture	100
Henry GB et al., [25]	2012 (Gray)	Nigeria	CS	505	0	Urine and Blood	Microscope+culture	100
Lam PM et al., [16]	2006	Nigeria	CS	410	6	Urine and Stool	Microscope+Culture	100
Mohager MO et al., [17]	2014	Sudan	CS	288	36	Urine and Stool	Microscope+Culture	100
Nury WM et al., [26]	2022 (Gray)	Sudan	CS	100	52	Urine and Blood	Microscope+ICT	100
Salem AK et al., [6]	2015	Sudan	CS	203	23	Urine, Stool and Blood	Microscope+Culture	100
Adetayo.O et al., [27]	2025	Nigeria	CS	360	108	Urine	Microscope+ PCR+Culture	100

YP-Year of publication, SD-Study design, CS- Cross sectional study, SZ- Sample size, P-Total positive, SU-Specimen used, LM-Laboratory method used, QS-Quality appraisal.

#### 3.1.1. Prevalence of Schistosoma-Salmonella co-infections in Africa.

Among the 2,243 study participants from 7 articles, the pooled prevalence of Schistosoma-Salmonella co-infections was 9% [2−15%] at the 95%-CI according to the random effects model (Fig 2).

**Fig 2:**
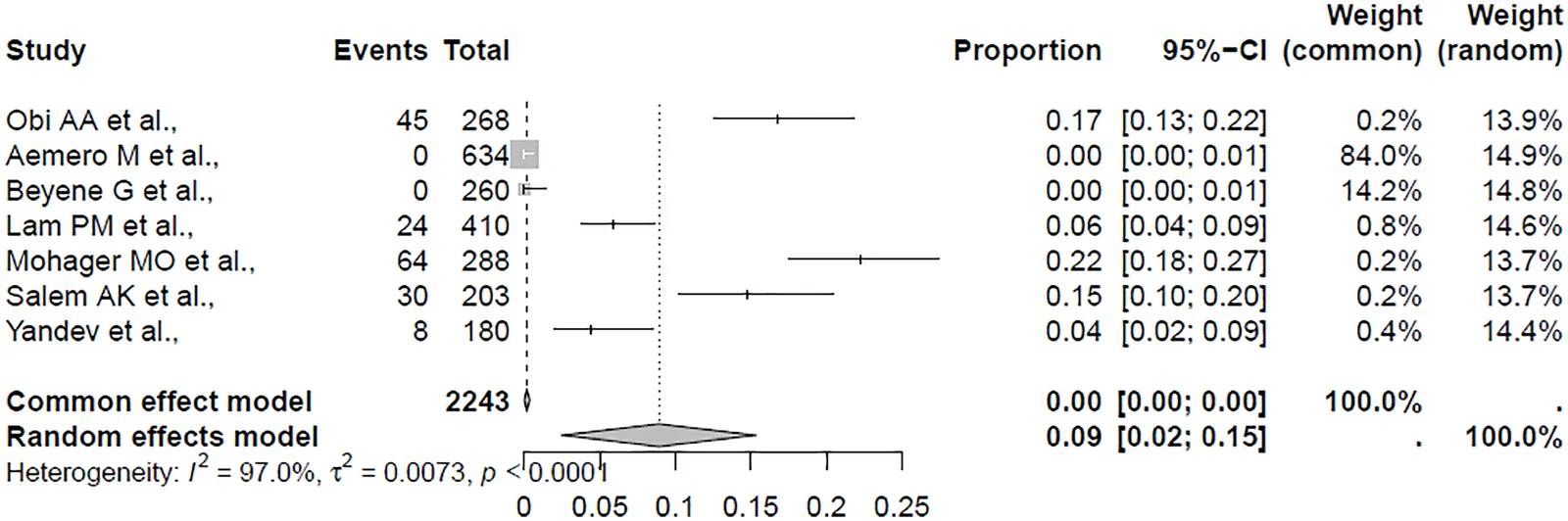
Prevalence of Schistosoma-Salmonella co-infections in Africa.

#### 3.1.2. Prevalence of *Schistosoma mansoni*-Salmonella co-infections in Africa.

Among the 3,999 study participants from 10 articles, the pooled prevalence of *Schistosoma mansoni*-Salmonella co-infections was 4% [1–6%] at the 95%-CI, P value <0.0001 according to the random effects model (Fig 3). Subgroup analysis by country was also performed, with a prevalence of 2% in Ethiopia, 9% in Sudan, 2% in Democratic republic of Congo, and 4% in Nigeria (Fig 4).

**Fig 3:**
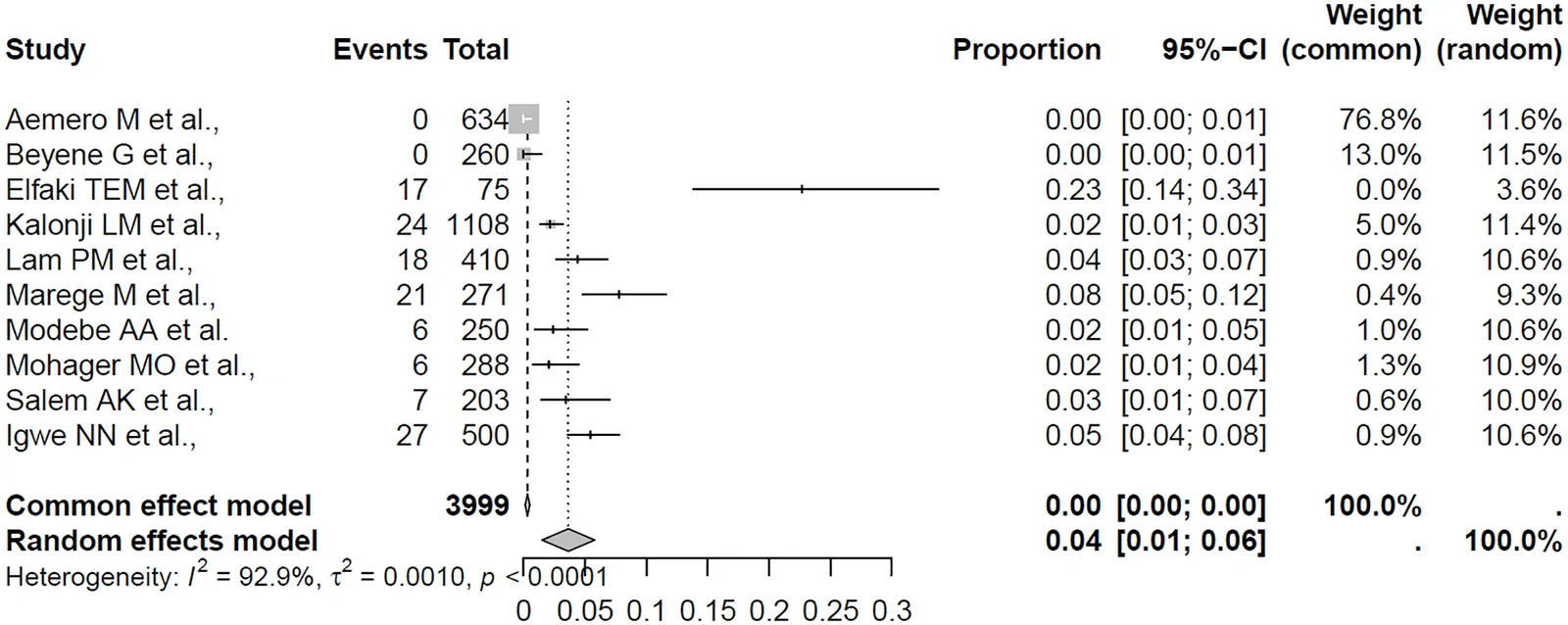
Prevalence of *Schistosoma mansoni*-Salmonella co-infections in Africa.

**Fig 4:**
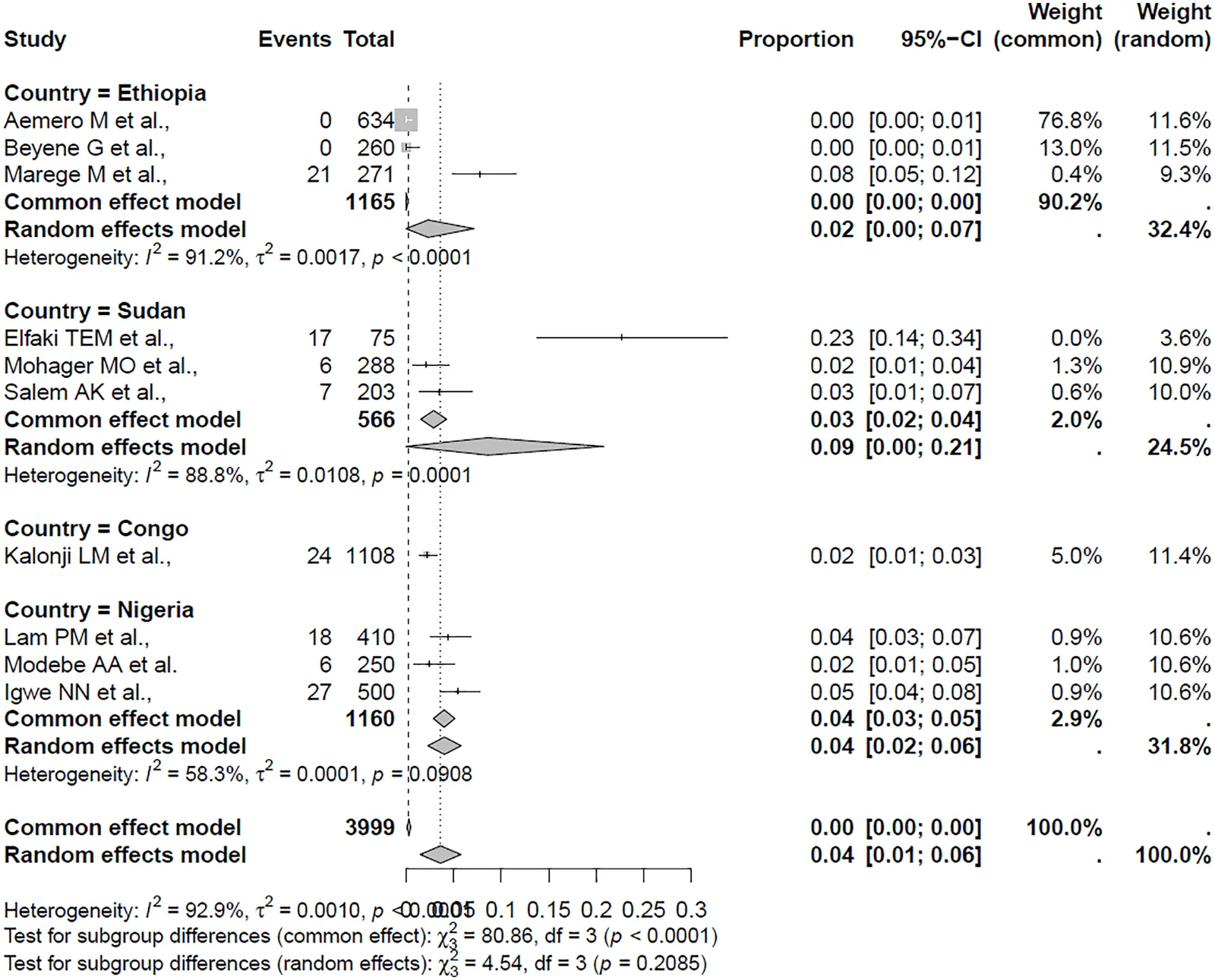
Subgroup analysis of *Schistosoma mansoni*-Salmonella co-infections in Africa.

#### 3.1.3. Prevalence of *Schistosoma hematobium*-Salmonella co-infections in Africa.

Among the 3170 study participants from 9 articles, the pooled prevalence of *Schistosoma hematobium*-Salmonella co-infections was 12% [0–23%] at the 95%-CI according to the random effects model (Fig 5). Subgroup analysis by country was also performed, with a prevalence of 0% in Ethiopia, 25% in Sudan, and 8% in Nigeria (Fig 6).

**Fig 5:**
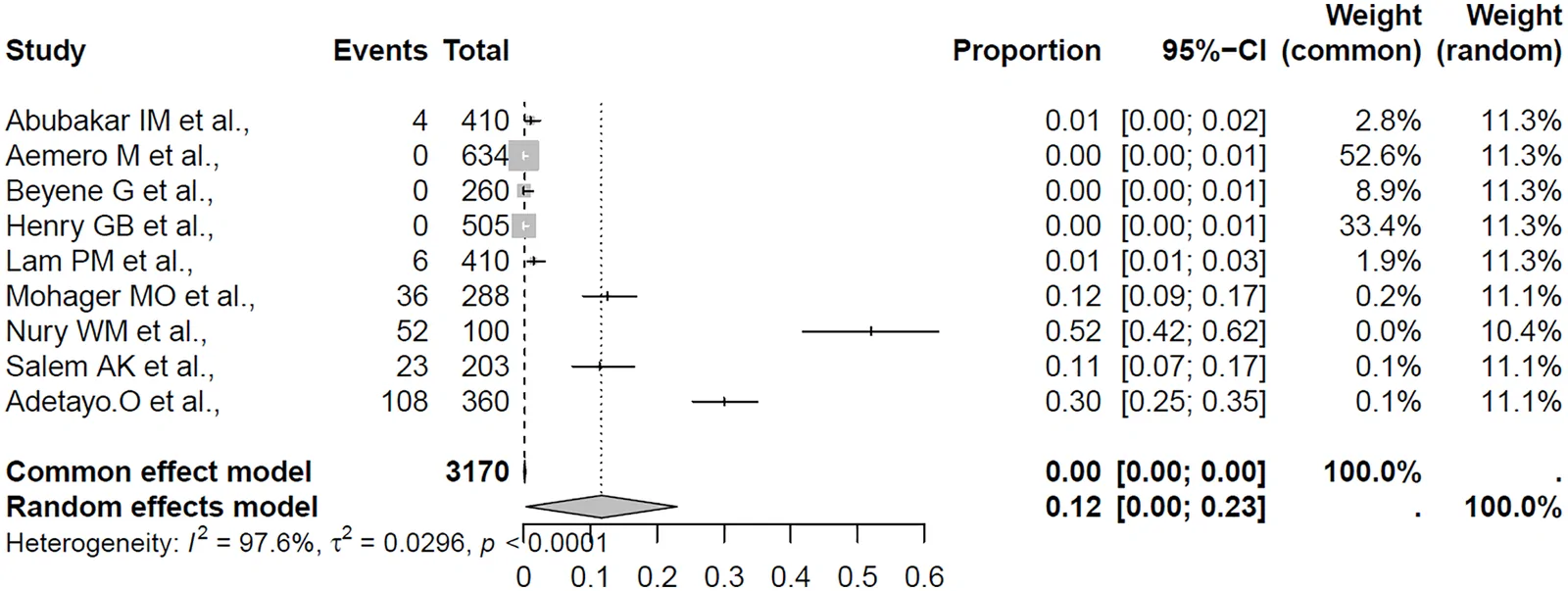
Prevalence of *Schistosoma hematobium*-Salmonella co-infections in Africa.

**Fig 6:**
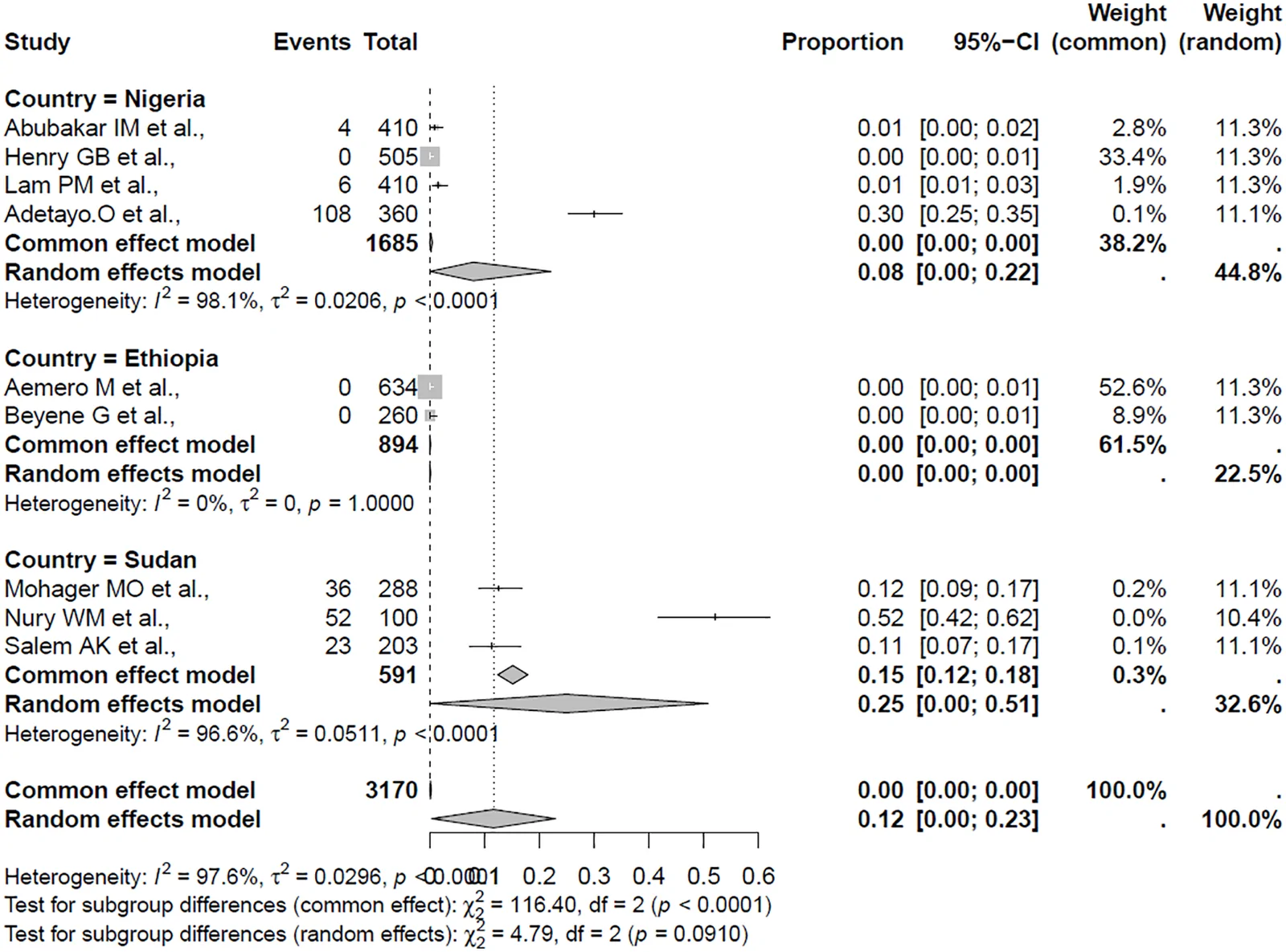
Subgroup analysis of *Schistosoma hematobium*-Salmonella co-infections in Africa.

### 3.2. Overview of the narrative review

#### 3.2.1. Biological mechanisms of Schistosoma-Salmonella Co-infection.

Schistosoma-Salmonella co-infection results from interconnected mechanisms involving immune modulation, tissue pathology, and direct pathogen-pathogen interactions, leading to persistent and often treatment-refractory infection [1]. Both infections can impair hepatic and renal function, potentially altering host metabolic processes and the pharmacokinetics and pharmacodynamics of therapeutic agents. In chronic schistosomiasis, eggs become trapped in tissues, particularly the liver and other organs, where they release soluble egg antigens and proteolytic enzymes that induce persistent granulomatous inflammation [28]. Over time, this result in fibrosis and structural damage, creating a micro-environment that facilitates secondary bacterial infections, including Salmonella [1].

The central mechanism is schistosome-driven immune modulation. Experimental studies demonstrated that, Schistosome infection induces a dominant Th2 immune response characterized by increased IL-4 and IL-13 production, IgE elevation, and eosinophilia, while suppressing pro-inflammatory and Th1/Th17 cytokines [29]. This shift promotes alternatively activated (M2) macrophages and reduces effective intracellular killing mechanisms required for Salmonella clearance [29]. Further amplification of this response occurs through schistosome egg-derived immunomodulatory molecules such as IPSE/α-1, which stimulate basophils to release IL-4 and IL-13, reinforcing Th2 polarization and suppressing Th1/Th17-mediated antibacterial immunity [3,22,30]. Additionally, expansion of regulatory T and B cells further contributes to immune suppression and bacterial persistence [30].

Direct physical interaction between the pathogens also plays a key role. Evidence for this mechanism is predominantly experimental. Salmonella can colonize the tegument and intestinal tract of adult Schistosomes via fimbrial adhesins (FimH) binding to mannose receptor-like glycol-proteins on the worm surface [31]. This interaction creates a protected intravascular niche that supports bacterial persistence and replication while reducing immune and antibiotic accessibility [20,22,30]. Experimental studies confirm increased bacterial burden and impaired macrophage bactericidal activity in schistosome-infected hosts, further enhancing systemic dissemination of Salmonella [3,20]. Clinically, co-infection is associated with chronic septicemic salmonellosis characterized by persistent bacteremia, hepatosplenomegaly, and recurrent febrile illness [17,22,30]. Treatment failure is common when Salmonella infection is managed without addressing underlying schistosomiasis, as bacteria protected within or attached to worms may persist despite antibiotic therapy [21,22]. However, the specific contribution of FimH-mediated attachment or worm-associated bacterial persistence mechanisms to treatment failure in humans remains to be definitively demonstrated.

Although most evidence indicates that schistosomiasis promotes Salmonella persistence through immune modulation and physical shielding, experimental studies also demonstrated a bidirectional interaction. Salmonella typhimurium infection has been shown to reduce Schistosoma japonicum worm and egg burdens, ameliorate disease pathology, and partially restore metabolic disturbances through inverse immune polarization [32]. These findings suggest that the interaction between Schistosoma and Salmonella is bidirectional and highly dependent on the host immune response, parasite species, bacterial serovar, and stage of infection. These findings were obtained from experimental models and therefore provide evidence of a potential biological interaction rather than confirmation of the same effect in human co-infection

Experimental evidence further indicates that these interactions are dynamic over time and depend on the stage of schistosomiasis. During acute schistosomiasis, Salmonella typhimurium is associated with increased fecal shedding and reduced hepatic and splenic colonization, as well as decreased schistosome egg deposition. In contrast, chronic infection favors bacterial retention within hepatic and splenic tissues with reduced stool excretion, supporting persistent intravascular infection. Co-infection also modulates schistosomiasis-associated pathology by enhancing splenic inflammation and apoptosis while unexpectedly reducing collagen deposition and fibrosis [33]. These temporal and pathological effects have primarily been demonstrated experimentally, and their relevance to the clinical course of human Schistosoma-Salmonella co-infection requires further investigation.

#### 3.2.2. Antimicrobial resistance and treatment failure in co-infection.

A major clinical consequence of Schistosoma-Salmonella interaction is reduced antibiotic efficacy and persistent infection [1]. Salmonella attached to Schistosomes via fimbrial adhesins may be physically shielded from multiple antibiotic classes, thereby reducing drug susceptibility and enhancing bacterial persistence [20,21,30]. In this protected niche, bacteria may be exposed to sub-therapeutic antibiotic concentrations during treatment, creating selective pressure that may favor the emergence of a resistant population [21,30]. Therapeutic failure is further exacerbated by Schistosome induced immune suppression, which impairs bacterial clearance even in the presence of effective antibiotic therapy. In addition, anti-Schistosoma treatment may release previously protected bacteria into circulation following worm death, potentially contributing to recurrent bacteremia if not combined with appropriate antibacterial therapy [22,30]. Clinically nonspecific febrile presentations and overlapping symptoms contribute to delayed or misdiagnosis, increasing inappropriate antibiotic exposure and further complicating treatment outcomes [22].

Experimental antibiotic protection assays have demonstrated that Schistosoma-associated Salmonella exhibit a 4–16-fold reduction in susceptibility to eight commonly used antibiotics, mediated by FimH-dependent binding of the bacteria to the Schistosoma tegument [31].

## 4. Discussion

Schistosoma-Salmonella co-infection is an important public health problem and a major challenge to clinical management in endemic areas [1]. A recent study in co-infected patients suggests a symbiotic relationship between the bacteria and the parasite [20]. This co-infection occurs when invasive Salmonella adheres to adult Schistosoma, resulting in persistent infection and various clinical manifestations [5]. Untreated schistosomiasis may lead to recurrent bacteremia following Salmonella therapy [34]. In addition, latent bacteria within Schistosoma reduce antibiotic effectiveness and may contribute to drug resistance [35]. Co-infection is common in Africa [22], particularly among schoolchildren [36], yet its prevalence and distribution across endemic regions remain insufficiently studied.

This systematic review synthesized the prevalence of Schistosoma-Salmonella co-infections, *Schistosoma mansoni*-Salmonella co-infections, *Schistosoma hematobium*-Salmonella co-infections, biological mechanisms and antimicrobial resistance of the co-infections in Africa. This study highlights a concise report on co-infection, and it benefits clinicians, epidemiologists and policymakers for evidence-based management. It will also be important for local and regional health policymakers to design appropriate methods of prevention and control in Africa. To date, a co-infection report in Africa is limited and this is the first systematic review study that addresses the epidemiology, biological mechanisms, and antimicrobial resistance of Schistosoma-Salmonella co-infections in Africa.

This updated systematic review on recent observational data of all the available literature on the prevalence of Schistosoma-Salmonella, *Schistosoma mansoni*-Salmonella and *Schistosoma hematobium*-Salmonella co-infection included 16 records in Africa (Fig 1). Among these records, 7 eligible articles are on Schistosoma Salmonella co-infection with pooled prevalence was 9%, 95%-CI [2−15%] (Fig 2). This figure is lower than studies conducted in Sudan (76%) [37], in Nigeria (16.7%) [13], and Sudan (22.2%, 14.7%) [6,17], and higher than the studies conducted in Nigeria (5.8%, 4.4%) [18,33] and Ethiopia (0%,0%) [14,15]. This variation could be due to sample size variation, diagnostic methods, study populations, and sampling season.

Besides, of the 16 records, 10 articles are on *Schistosoma mansoni*-Salmonella co-infection with pooled prevalence was 4%, 95-CI [1–6%] (Fig 3). This figure is comparable with studies reported from Nigeria (4.4%, 5.4%) [23,33] and Sudan (3.4%) [6] and lower than reports from Egypt (9%) [38], Ethiopia (7.7%) [21] and Sudan (22.7%) [19], and higher than studies conducted in Ethiopia (0%, 0%) [14,15], Congo (2.2%) [20], Nigeria (2.4%) [22] and Sudan (2.1%) [17]. This might be due to differences in the endemicity and transmission intensity of *Schistosoma mansoni* across study areas, variations in exposure to contaminated freshwater, sanitation conditions, and infection intensity may contribute to the observed heterogeneity [2,39,40].

Similarly, subgroup analysis of the prevalence of *Schistosoma mansoni*-Salmonella co-infection was performed by country as follows: 2% in Ethiopia, 9% in Sudan, 4% in Nigeria and 2% in Congo (Fig 4). This observed country level heterogeneity may be due to the differences in *Schistosoma mansoni* endemicity and transmission intensity and implementation of schistosomiasis control programs across Ethiopia, Sudan, Nigeria, and Congo [2,39].

Moreover, out of 16 records, 9 eligible primary articles were on *Schistosoma hematobium*-Salmonella co-infection with an overall prevalence was 12% [0–23%] (Fig 5). This is comparable with report from Sudan (12.5%) [17] and lower than studies conducted in Nigeria (30%) [27] and Sudan (52%) [26] but higher than studies conducted in Ethiopia (0%,0%) [14,15], Nigeria (0%) [25], Nigeria (1.46%) [33] and Sudan (11.3%) [6].

This might be due to variation in control interventions and the endemicity and transmission intensity of urinary Schistosomiasis in different settings, diagnostic modalities used to detect *Schistosoma* and *Salmonella* infections, study populations, and differences in sampling frameworks, study settings, sample sizes, and local epidemiological conditions could be the possible reason for variation [39,41].

Similarly, subgroup analysis on the prevalence of *Schistosoma hematobium*-Salmonella co-infection was performed by country as follows: 0% in Ethiopia, 25% in Sudan, and 8% in Nigeria (Fig 6). This country level heterogeneity might be due to differences in the implementation control programs, chronic urinary tract pathology caused by *Schistosoma haematobium* infection and endemicity and transmission intensity of urinary schistosomiasis across Ethiopia, Sudan, and Nigeria [39,41].

Evidence synthesized in this narrative review indicates that Schistosoma-Salmonella co-infection is driven by immune modulation, tissue pathology, and direct pathogen interactions, which could contribute to bacterial persistence. These mechanisms may have important clinical implications particularly in endemic areas where co-infection may complicate diagnosis and management. However, the current evidence is derived predominantly from experimental and mechanistic studies, with limited confirmation from well-designed human studies. Hence, the extent to which these mechanisms contribute to persistent infection or clinical outcomes in human remains uncertain. Future research should prioritize prospective clinical and translational studies to clarify the underlying mechanisms and evaluate their implications for diagnosis, treatment, and control of Schistosoma Salmonella co-infection.

The available evidence also suggests that treatment failure in Schistosoma-Salmonella co-infection may not be explained solely by antimicrobial resistance, that host and tissue factors associated with schistosomiasis may contribute to bacterial persistence and reduced treatment effectiveness [1]. However, the current evidence is insufficient to establish that bacterial persistence is the primary driver of treatment failure or to distinguish its contribution from antimicrobial resistance. Thus, these mechanisms should be considered potential contributors rather than established causes of treatment failure. Further clinical studies are needed to determine the relative contributions of antimicrobial resistance, bacterial persistence, host immune modulation, and tissue pathology to treatment outcomes in endemic populations.

## 5. Conclusions

This review highlights Schistosoma-Salmonella co-infection as an overlooked public health concern in Africa, the available evidence indicates that this neglected co-infection is characterized by a considerable epidemiological burden, complex biological interactions, and important therapeutic challenges. Strengthened surveillance, early diagnosis, integrated clinical management, and further research are essential to improve control and patient outcome in endemic settings.

### 5.1. Limitations of the study

Only studies published in English languages and studies retrieved from open access sources were used in the analysis, which may introduce language bias and have led to the exclusion of relevant subscription-based articles. Besides, protocol registration was not undertaken as review was initiated before prospective protocol registration. Moreover, due to the limited number of studies addressing the biological mechanisms and antimicrobial resistance of *Schistosoma-Salmonella* co-infections, these aspects could not be quantitatively synthesized and were therefore assessed narratively.

## Supporting information

S1 File PRISMA 2020 Checklist. (DOCX)

S2 File JBI Checklist, 2017 for cross-sectional studies. (DOCX)

## Abbreviations

CDC: center of disease control
CI: confident interval
CINAHL: cumulative index to nursing and allied health literature
df: degree of freedom
Fimh: Fimbrial adhesins
MOH: ministry of health
IgE: immunoglobulin- E
IL: interleukin
IPSE/α-1: interleukin principle Schistosoma egg alpha-1 isomer
MeSH: medical science heading
Th: T-helper cell
PRISMA-P: preferred reporting items for systemic review and meta-analysis protocols
S-1: supplementary file one- PRISMA checklist
S-2: supplementary file two-quality appraisal JBI checklist for Schistosoma-Salmonella co-infection
SE: standard error
WHO: World Health Organization
