# Supplementary material for: Schistosoma-Salmonella co-infection in Africa: Epidemiology, biological mechanisms, and antimicrobial resistance: A systematic review and meta analysis
Source: PLoS One. 2026 Oct 29;21(10):e0359975. doi: 10.1371/journal.pone.0359975 (PMC13718923; doi:10.1371/journal.pone.0359975)
Supplement: Supplementary file 1 [file pone.0359975.s003.pdf]

PLOS One

Schistosoma -Salmonella co-infection in Africa: Epidemiology, Biological mechanisms, and Antimicrobial resistance. A systematic review and Meta analysis

--Manuscript Draft--

**Manuscript Number:**	PONE-D-26-36772
**Article Type:**	Research Article
**Full Title:**	Schistosoma -Salmonella co-infection in Africa: Epidemiology, Biological mechanisms, and Antimicrobial resistance. A systematic review and Meta analysis
**Short Title:**	Schistosoma-Salmonella co-infection in Africa
**Corresponding Author:**	Brhane Berhe Aregawi, MSc<br>Adigrat University College of Health Sciences<br>Adigrat, Tigrai ETHIOPIA
**Keywords:**	Antimicrobial resistance, Biological Mechanism, co-infection, Incidence, Prevalence, Salmonella, Schistosoma
**Abstract:**	Background: Schistosomiasis and salmonellosis are major public health problems in Africa, contribute substantially to morbidity. Schistosoma-Salmonella co-infection is a neglected and overlooked health condition with important clinical and public health implications. Therefore, we conducted a systemic review and meta-analysis to synthesize evidence on the epidemiology, biological mechanisms, and antimicrobial resistance of Schistosoma-Salmonella co-infection in Africa.<br>Methods: A systematic review and Meta-analysis were conducted following the PRISMA framework. R statistical software version 4.5.1 was used to obtain the pooled prevalence and odds ratio via a random effects model.<br>Results: This systemic review found that the pooled prevalence of Schistosoma-Salmonella co-infection in Africa was 9%. Species specific pooled prevalence was 4% for Schistosoma mansoni-Salmonella co-infection and 12% for Schistosoma hematobium-Salmonella co-infection. Country based subgroup analysis showed prevalence of Schistosoma mansoni-Salmonella co-infection of 2% in Ethiopia, 9% in Sudan, 2% in Democratic republic of Congo and 4% in Nigeria, while Schistosoma hematobium-Salmonella co-infection prevalence were 0%, 25%, and 8% in Ethiopia, Sudan, and Nigeria respectively. Schistosoma-Salmonella co-infection is driven by immune modulation, tissue pathology, and parasite bacterium interactions that support persistent infection and compromise therapeutic efficacy, thereby contributing to treatment failure and the emergence of antimicrobial resistance.<br>Conclusions : Schistosoma-Salmonella co-infection represents an important and under recognized public health concern in Africa, with a pooled prevalence of 9%. The findings demonstrate notable variation in co-infection prevalence across Schistosoma species and geographical settings, with higher rates observed among Schistosoma haematobium compared with Schistosoma mansoni. Strengthened surveillance systems, improving early diagnosis, and implementing integrated control strategies are essential to reduce the burden and enhance clinical management of this neglected co-infection in Africa.
**Order of Authors:**	Brhane Berhe Aregawi, MSc
	Hadush Negash
	Haftom Legesse
	Getachew Belay
	Abel Deres
	Merhawi Alemu
	Aberham Abere
**Additional Information:**	
**Question**	**Response**

Powered by Editorial Manager® and ProduXion Manager® from Aries Systems Corporation

**Financial Disclosure**<br><br>Enter a financial disclosure statement that describes the sources of funding for the work included in this submission. Review the submission guidelines for detailed requirements. View published research articles from *PLOS ONE* for specific examples.<br><br>This statement is required for submission and **will appear in the published article** if the submission is accepted. Please make sure it is accurate.<br><br>**Funded studies**<br>Enter a statement with the following details:<br>• Initials of the authors who received each award<br>• Grant numbers awarded to each author<br>• The full name of each funder<br>• URL of each funder website<br>• Did the sponsors or funders play any role in the study design, data collection and analysis, decision to publish, or preparation of the manuscript?<br><br>Did you receive funding for this work?	The author(s) received no specific funding for this work.
**Competing Interests**<br><br>Use the instructions below to enter a competing interest statement for this submission. On behalf of all authors, disclose any competing interests that could be perceived to bias this work—acknowledging all financial support and any other relevant financial or non-financial competing interests.<br><br>This statement is required for submission and **will appear in the published article** if the submission is accepted. Please make sure it is accurate and that any funding sources listed in your Funding Information later in the submission form are also declared in your Financial Disclosure statement.	The authors have declared that no competing interests exist.

Powered by Editorial Manager® and ProduXion Manager® from Aries Systems Corporation

View published research articles from *PLOS ONE* for specific examples.<br><br>**NO authors have competing interests**<br><br>Enter: *The authors have declared that no competing interests exist.*<br><br>**Authors with competing interests**<br><br>Enter competing interest details beginning with this statement:<br><br>*I have read the journal's policy and the authors of this manuscript have the following competing interests: [insert competing interests here]*<br><br>* typeset	
**Ethics Statement**<br><br>Enter an ethics statement for this submission. This statement is required if the study involved:<br><br>• Human participants<br>• Human specimens or tissue<br>• Vertebrate animals or cephalopods<br>• Vertebrate embryos or tissues<br>• Field research<br><br>Write "N/A" if the submission does not require an ethics statement.<br><br>General guidance is provided below. Consult the submission guidelines for detailed instructions. **Make sure that all information entered here is included in the Methods section of the manuscript.**	N/A

**Format for specific study types**<br><br>**Human Subject Research (involving human participants and/or tissue)**<br>• Give the name of the institutional review board or ethics committee that approved the study<br>• Include the approval number and/or a statement indicating approval of this research<br>• Indicate the form of consent obtained (written/oral) or the reason that consent was not obtained (e.g. the data were analyzed anonymously)<br><br>**Animal Research (involving vertebrate animals, embryos or tissues)**<br>• Provide the name of the Institutional Animal Care and Use Committee (IACUC) or other relevant ethics board that reviewed the study protocol, and indicate whether they approved this research or granted a formal waiver of ethical approval<br>• Include an approval number if one was obtained<br>• If the study involved *non-human primates*, add *additional details* about animal welfare and steps taken to ameliorate suffering<br>• If anesthesia, euthanasia, or any kind of animal sacrifice is part of the study, include briefly which substances and/or methods were applied<br><br>**Field Research**<br><br>Include the following details if this study involves the collection of plant, animal, or other materials from a natural setting:<br>• Field permit number<br>• Name of the institution or relevant body that granted permission	
**Data Availability**<br><br>Before publication, Authors are required to make fully available and without restriction all data underlying their findings. Sharing the data underlying your findings increases the visibility, credibility, and impact of your work, while supporting transparency and future scientific discovery. Please see our PLOS Data	The raw data used for this study will be made available upon request.

Policy page for detailed information on this policy.<br><br>A **Data Availability Statement**, detailing where the data can be accessed, is required at first submission. Insert your Data Availability Statement in the box below.<br><br>Please see the data reporting section of our submission guidelines for instructions on what you need to include in your Data Availability Statement. Examples of acceptable Data Availability Statements are included here for reference:<br>1. The minimal data set [and accompanying code, where generated] is available at [repository name] via [DOI hyperlink]<br>2. No data was generated by this study. The following existing data sources were used: [dataset name] from [dataset location] available via [web address or DOI hyperlink].<br>3. For Study Protocols: No datasets were generated or analysed during the current study. All relevant data from this study will be made available upon study completion.<br><br>*PLOS allows rare exemptions to address legal and ethical concerns. If you have legal or ethical restrictions, please use the box below to detail these in full sentences for the Journal team to consider.*<br><br>This statement will be typeset if the manuscript is accepted for publication.	

Powered by Editorial Manager® and ProduXion Manager® from Aries Systems Corporation

Final Manuscript

Click here to access/download;Manuscript;Final Manuscript on Schistosomiasis-Salmonella Co-infection.docx

Schistosoma-Salmonella co-infection in Africa: Epidemiology, Biological mechanisms, and Antimicrobial resistance. A systematic review and Meta analysis

Brhane Berhe*[1], Hadush Negash[1], Tesfay Abadi[1], Getachew Belay[1], Merhawi Alemu[1], Aberham Abere[2]

Haftom Legese[1], Abel Deres [1]

[1]Department of Medical Laboratory Science, College of Medicine and Health Sciences, Adigrat, Ethiopia, P.O. Box 50

[1]Department of Medical Parasitology, School of Biomedical and Laboratory Sciences, College of Medicine and Health Sciences, University of Gondar, Gondar, Ethiopia, P.O. Box 196

BB: birhaneberhe54@gmail.com
HN: hadunegash@gmail.com
HL: legesehaftom2@gmail.com
GB: Getabelay65@gmail.com
AD: abelderes.wediraya@gmail.com
TA: tesfayab3@gmail.com
MA: alemumerhawi21@gmail.com
AA: aberhamaber21@gmail.com

Correspondence

Brhane Berhe Aregawi, MSc

Adigrat University College Medicine and health Science,
Department of Medical laboratory Science,
Adigrat, Ethiopia, P.O. Box: 50
birhaneberhe54@gmail.com

Abstract

Background: Schistosomiasis and salmonellosis are major public health problems in Africa, contribute substantially to morbidity. *Schistosoma-Salmonella* co-infection is a neglected and overlooked health condition with important clinical and public health implications. Therefore, we conducted a systemic review and meta-analysis to synthesize evidence on the epidemiology, biological mechanisms, and antimicrobial resistance of *Schistosoma-Salmonella* co-infection in Africa.

Methods: A systematic review and Meta-analysis were conducted following the PRISMA framework. R statistical software version 4.5.1 was used to obtain the pooled prevalence and odds ratio via a random effects model.

Results: This systemic review found that the pooled prevalence of *Schistosoma-Salmonella* co-infection in Africa was 9%. Species specific pooled prevalence was 4% for *Schistosoma mansoni-Salmonella co-infection* and 12% for *Schistosoma hematobium-Salmonella* co-infection. Country based subgroup analysis showed prevalence of *Schistosoma mansoni-Salmonella* co-infection of 2% in Ethiopia, 9% in Sudan, 2% in Democratic republic of Congo and 4% in Nigeria, while *Schistosoma hematobium-Salmonella* co-infection prevalence were 0%, 25%, and 8% in Ethiopia, Sudan, and Nigeria respectively. *Schistosoma-Salmonella* co-infection is driven by immune modulation, tissue pathology, and parasite bacterium interactions that support persistent infection and compromise therapeutic efficacy, thereby contributing to treatment failure and the emergence of antimicrobial resistance.

Conclusions: Schistosoma-*Salmonella* co-infection represents an important and under-recognized public health concern in Africa, with a pooled prevalence of 9%. The findings demonstrate notable variation in co-infection prevalence across *Schistosoma* species and geographical settings, with higher rates observed among *Schistosoma haematobium* compared with *Schistosoma mansoni*. Strengthened surveillance systems, improving early diagnosis, and implementing integrated control strategies are essential to reduce the burden and enhance clinical management of this neglected co-infection in Africa.

Keywords: Antimicrobial resistance, Biological Mechanism, co-infection, Incidence, Prevalence, Salmonella, Schistosoma

1. Background and Justification

Schistosomiasis is a neglected tropical disease caused by blood flukes, affecting over 250 million people worldwide, with about 90% of cases occur in Africa (1). In Africa, the disease is mainly due to *Schistosoma mansoni* and *Schistosoma haematobium* (1, 2).

Salmonellosis is another important infectious disease causing a significant morbidity and mortality in Africa and remains a major cause of bloodstream and gastrointestinal infections particularly in children (3). The burden is aggravated by delayed diagnosis, poor laboratory infrastructure, and increasing antimicrobial resistance (4).

Schistosomiasis and salmonellosis coexist in many African countries where both diseases remain endemic, yet *Schistosoma-Salmonella* co-infection remains poorly understood (1, 3). Geographic distribution, variation in control program, inadequate sanitation, and unsafe water sources are the contributing factors for persistent of the co-infection (1, 2). However, reports showed variation in prevalence across African countries (5, 6).

The biological interaction mechanisms underlying Schistosoma-*Salmonella* co-infection remain a major challenge. Previous studies have demonstrated that *Salmonella* organisms can attach to adult *Schistosoma* worms, enhancing bacterial survival by facilitating immune evasion and reducing susceptibility to antimicrobial therapy (5). This interaction may contribute to prolonged infection and increased disease burden, particularly in endemic regions of Africa (6). However, in *Schistosoma*- endemic area co-infections are likely underreported due to limited diagnostic capacity, insufficient clinical awareness, and overlapping clinical manifestations.

Antimicrobial resistance of *Salmonella* is an emerging public health concern in Africa and poses additional challenges for effective treatment (4). The presence of *Schistosoma-Salmonella* co-infection may further compromise therapeutic outcomes by promoting bacterial persistence, immune evasion and recurrent infection (5). However, comprehensive evidence regarding the epidemiology, biological mechanisms, and antimicrobial resistance of *Schistosoma-Salmonella* co-infection remains limited. This knowledge gap hinders clinical management, public health surveillance, and the

development of effective control strategies. Therefore, we aimed to conduct a systematic review and meta-analysis of the epidemiology of *Schistosoma-Salmonella* co-infection in Africa, and synthesize evidence on its biological mechanisms, and antimicrobial resistance implications.

2. Materials and methods

2.1. Study setting

This systematic review was conducted to study the epidemiology, biological mechanisms, and antimicrobial resistance of *Schistosoma-Salmonella* co-infection in Africa, with a geographical area of 30.37 million km^2 and an over 1.56 billion people, the second largest continent in the world next to Asia (7).

2.2. Protocol design

This systematic review summarized evidence exclusively observational studies (cross-sectional) that were conducted on the basis of studies reported across different regions in Africa. It was performed in accordance with the Preferred Reporting Items for Systematic Reviews and Meta-analyses Protocol (PRISMA-P) (S-1) and checklist [S-2] (8) and meta-analysis of observational studies in epidemiology guidelines (9). Despite the PRISMA-P checklist was used, a formal review protocol was not registered

2.3. Search strategies and sources

A search strategy was developed using specific key concepts in our research questions, such as the Prevalence, Epidemiology, Biological mechanism, Antimicrobial resistance of *Schistosoma Salmonella* co-infection in Africa. For each key concept, appropriate free-text words and Medical Subject Headings (MeSH) were developed. The free-text words (truncated or with wildcards) and MeSH terms were combined via Boolean logic operators: “AND” and “OR”. We comprehensively searched various databases, including PubMed, Google Scholar, EMBASE, Cochrane, Global Health and CINAHL. The search was performed without date restrictions, and the final search was conducted on July 5, 2026. Only studies published in English were considered. The search strategy for each database was independently validated by three authors. We used the following search words: (((("Prevalence"[Mesh]) OR "Epidemiology"[Mesh]) OR “Biological mechanism” AND "Schistosoma *Salmonella* co-infection"[Mesh]) OR "Antimicrobial resistance” [Mesh]))))). We also used keywords such as “Prevalence and

Schistosoma mansoni co-infection in Africa", "Epidemiology and *Schistosoma mansoni Salmonella* co-infection in Africa", "Incidence and *Schistosoma mansoni salmonella* co-infection in Africa", "Prevalence and *Schistosoma haematobium Salmonella* co-infection in Africa", "Epidemiology and *Schistosoma haematobium Salmonella* co-infection in Africa", and "Incidence and Urinary schistosomiasis *Salmonella* co-infection in Africa".

To avoid missing relevant studies and gray literature, we searched other sources by looking through reference lists of relevant reviews and selected studies, searching websites of a relevant organization (WHO, CDC, FMOH), and searching for relevant articles via PubMed-related article features, Google Scholar, the World Health Organization and Centers for Disease Control and Prevention websites, and proceedings of the International Union Against Schistosomiasis Conference proceedings (Conference Proceedings Citation Index-Science (CPCI-S)), Dissertations & Thesis (www.proquest.com).

2.4. Study selection and data extraction

After the literature search, all the references were imported to EndNote X7.8 (Bid 7705). The collected data were purposefully processed via Microsoft Excel. Two researchers (BB and HL) independently screened studies for eligibility. Following screening, studies were selected irrespective of their design and time if they met the inclusion criteria. The researchers autonomously reviewed titles and abstracts against eligibility criteria to categorize the article as either 'to include' or 'exclude'. A third researcher (AD) was consulted for discrepancies at each stage of the selection. We resolved differences in opinion through discussion. Data were extracted by BB and HL from each selected study via a predetermined list of categories, including first author, year of publication, Country, setting (community/health facility), study design, prevalence of *Schistosoma Salmonella* co-infection, prevalence of *Schistosoma mansoni Salmonella* co-infection, prevalence of *Schistosoma hematobium Salmonella* co-infection, type of specimen used, and laboratory method used, biological mechanism of *Schistosoma Salmonella* co-infection reported, antimicrobial resistance on *Schistosoma Salmonella* reported via Microsoft Excel.

2.5. Handling of missing data

We requested corresponding authors through electronic mail and research gate to provide missing outcome data for some studies.

2.6. Eligibility criteria

Articles with human subjects who were available through open access briefly demonstrating study selection and data extraction, had a quality assessment score of 50%, and were sufficiently relevant to satisfy the review selection criteria were included for the Meta analysis where as for the biological mechanisms and antimicrobial resistance pattern, human and animals subject were used to narrate the mechanisms.

2.7. Quality assessment and critical appraisal

The quality of each study included in the review was assessed via a critical appraisal tool adopted from the JBI checklist of 2017 to assess the validity and quality of studies, the recommended tool for evaluating primary studies for inclusion in systematic reviews for etiology and risk review (10). A third researcher (AD) was consulted to resolve discrepancies while appraising the quality assessment. The items used for the quality assessments are presented in S-2 and S-3.

2.8. Data analysis

R statistical software version 4.5.1 (Rx64 3.6.1, R-4.5.1-win.exe) was utilized to carry out the Meta-analysis. A Meta-analysis of proportions was conducted to estimate the pooled prevalence of *Schistosoma Salmonella* co-infection. Considering the expected heterogeneity across studies, a random-effects model was applied using the DerSimonian-Laird estimator for tau2 and the Clopper-Pearson confidence interval for individual studies. To alleviate the variance of proportions, a variance-stabilizing transformation was applied prior to pooling. Heterogeneity among studies was assessed using Cochran's Q test and quantified using the I^2 statistic. An I^2 value greater than 50% was considered indicative of substantial heterogeneity.

2.9. Sub-group analysis

Sub group analyses were carried to explore potential sources of heterogeneity based on study country. In addition, sensitivity analyses were performed to assess the robustness

of pooled estimates by sequentially excluding individual studies. Publication bias was not evaluated as the minimum criteria to undergo are 10 primary research articles.

2.10. Assessment of heterogeneity, and publication bias

We assessed the extent of heterogeneity among studies with forest plots with 95% prediction regions and statistically with chi-square (χ2), P value and I-square (I^2) tests (11). Publication bias for this systematic review was not done due to the study articles were less than 10 (12). Symmetricity was screened via a funnel plot test.

3. Results

3.1. Overview of the systematic and Meta analysis review

This systematic review identified 104 publication records through a database search. Among these records, 17 research articles on the prevalence of *Schistosoma-Salmonella* co-infections, 56 articles on the prevalence of *Schistosoma mansoni-Salmonella* co-infections, and 31 articles were on the prevalence of *Schistosoma hematobium-Salmonella* co-infections. After duplicates were removed, 11 studies on *Schistosoma-Salmonella* co-infections, 23 articles on *Schistosoma mansoni-Salmonella* co-infections and 15 studies articles on *Schistosoma hematobium-Salmonella* co-infections were screened on the basis of their titles and abstracts. Nine articles for the prevalence *Schistosoma-Salmonella* co-infections, 20 articles for *Schistosoma mansoni-Salmonella* co-infections and 10 articles for *Schistosoma hematobium-Salmonella* co-infections were assessed as eligible for full-text reviews. After a comprehensive reading, 7 articles on the prevalence of *Schistosoma-Salmonella* co-infections, 10 articles on the prevalence of *Schistosoma mansoni-Salmonella* co-infections and 9 studies on the prevalence of *Schistosoma haematobium-Salmonella* co-infections met the inclusion criteria and were included in this systemic review and meta-analysis. The list of studies included is provided in **S-4**. The review selection process was illustrated via a PRISMA diagram **(S-1)**.

S-4 (Table-1): Results on the prevalence of schistosomiasis-salmonella co-infections in Africa

Author	YP	Country	SD	SZ	P	SU	LM	QS (%)
Obi AA *et al*., (13)	1996	Nigeria	CS	268	45	Urine, Stool and Blood	Microscope+Widal	100
Aemero M *et al*.,(14)	2012	Ethiopia	CS	634	0	Urine and Stool	Microscope+Culture	100
Beyene G *et al*.,(15)	2014	Ethiopia	CS	260	0	Stool	Micrpscope+Culture	100
Lam PM *et al*.,(16)	2006	Nigeria	CS	410	24	Urine and Stool	Miscroscope+culture	100
Mohager MO *et al*.,(17)	2014	Sudan	CS	288	64	Urine and Stool	Microscope+Culture	100
Salem AK *et al*.,(6)	2015	Sudan	CS	203	30	Urine, Stool and Blood	Microscope+Culture	100
Yandev *et al*.,(18)	2022	Nigeria	CS	180	8	Urine and Stool	Microscope+Culture	100

YP-Year of publication, SD-Study design, CS- Cross sectional study, SZ- Sample size, P-Total positive, SU-Specimen used, LM-Laboratory method used, QS-Quality appraisal

S-4 (Table-2): Results on the prevalence of Schistosoma mansoni-salmonella co-infections in Africa

Author	YP	Country	SD	SZ	P	SU	LM	QS (%)
Aemero M *et al*.,(18)	2012	Ethiopia	CS	634	0	Urine and Stool	Microscope+Widal	100
Beyene G *et al*.,(15)	2014	Ethiopia	CS	260	0	Stool	Microscope+Culture	100
Elfaki TEM *et al*.,(19)	2015	Sudan	CS	75	17	Stool and Blood	Micrpscope+Culture	100
Kalonji LM *et al*.,(20)	2020	Congo	CS	1108	24	Stool and Blood	Miscroscope+culture	100
Lam PM *et al*.,(16)	2006	Nigeria	CS	410	18	Urine and Stool	Microscope+Culture	100
Marege M *et al*.,(21)	2021	Ethiopia	CS	271	21	Stool and Blood	Microscope+Culture	100
Modebe AA *et al*.(22)	2014	Nigeria	CS	250	6	Stool and Blood	Microscope+Widal+Culture	100
Mohager MO *et al*.,(17)	2014	Sudan	CS	288	6	Urine and Stool	Microscope+Culture	100
Salem AK *et al*.,(6)	2015	Sudan	CS	203	7	Urine, Stool and Blood	Microscope+Culture	100
Igwe NN *et al*.,(23)	2014	Nigeria	CS	500	27	Urine, Stool and Blood	Microscope+ICT	100

YP-Year of publication, SD-Study design, CS- Cross sectional study, SZ- Sample size, P-Total positive, SU-Specimen used, LM-Laboratory method used, QS-Quality appraisal

S-4 (Table-3): Results on the prevalence of Schistosoma hematobium-salmonella co-infections in Africa

Author	YP	Country	SD	SZ	P	SU	LM	QS (%)
Abubakar IM *et al*.,(24)	2025	Nigeria	CS	410	4	Urine and Stool	Microscope+Culture	100
Aemero M *et al*.,(14)	2012	Ethiopia	CS	634	0	Urine and Stool	Microscope+Culture	100
Beyene G *et al*.,(15)	2014	Ethiopia	CS	260	0	Stool	Micrpscope+Culture	100
Henry GB *et al*.,(25)	2012 (Gray)	Nigeria	CS	505	0	Urine and Blood	Miscroscope+culture	100
Lam PM *et al*.,(16)	2006	Nigeria	CS	410	6	Urine and Stool	Microscope+Culture	100
Mohager MO *et al*.,(17)	2014	Sudan	CS	288	36	Urine and Stool	Microscope+Culture	100
Nury WM *et al*.,(26)	2022 (Gray)	Sudan	CS	100	52	Urine and Blood	Microscope+ICT	100
Salem AK *et al*.,(6)	2015	Sudan	CS	203	23	Urine, Stool and Blood	Microscope+Culture	100
Adetayo.O *et al*., (27)	2025	Nigeria	CS	360	108	Urine	Microscope+ PCR+Culture	100

YP-Year of publication, SD-Study design, CS- Cross sectional study, SZ- Sample size, P-Total positive, SU-Specimen used, LM-Laboratory method used, QS-Quality appraisal

Prevalence of *Schistosoma-Salmonella* co-infections in Africa

Among the 2,243 study participants from 7 articles, the pooled prevalence of *Schistosoma-Salmonella* co-infections was 9% [2-15%] at the 95%-CI according to the random effects model **(Table-4).**

Table-4: Prevalence of *Schistosoma-Salmonella* co-infections in Africa

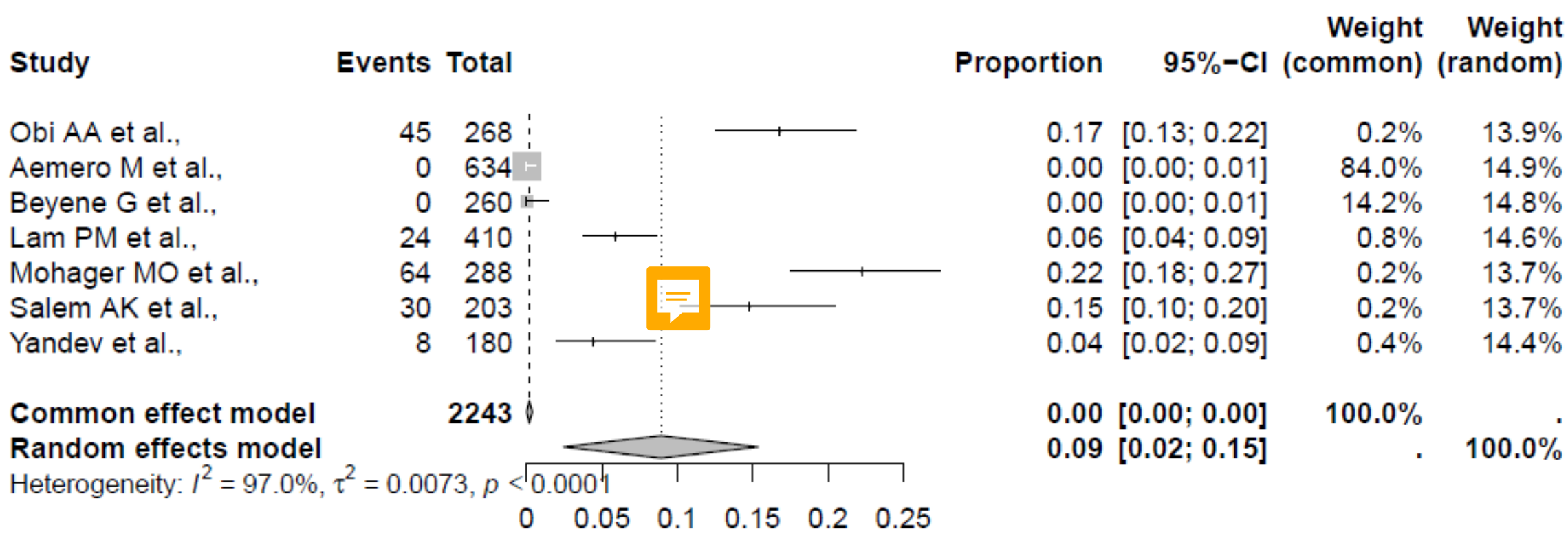

Prevalence of *Schistosoma mansoni-Salmonella* co-infections in Africa

Among the 3,999 study participants from 10 articles, the pooled prevalence of *Schistosoma mansoni-Salmonella* co-infections was 4% [1-6%] at the 95%-CI according to the random effects model **(Table-5).** Subgroup analysis by country was also performed, with a prevalence of 2% in Ethiopia, 9% in Sudan, 2% in Democratic republic of Congo, and 4% in Nigeria (**Table-6**).

Table-5: Prevalence of *Schistosoma mansoni-Salmonella* co-infections in Africa

Study	Events	Total	Proportion	95%−CI	Weight (common)	Weight (random)
Aemero M et al.,	0	634	0.00	[0.00; 0.01]	76.8%	11.6%
Beyene G et al.,	0	260	0.00	[0.00; 0.01]	13.0%	11.5%
Elfaki TEM et al.,	17	75	0.23	[0.14; 0.34]	0.0%	3.6%
Kalonji LM et al.,	24	1108	0.02	[0.01; 0.03]	5.0%	11.4%
Lam PM et al.,	18	410	0.04	[0.03; 0.07]	0.9%	10.6%
Marege M et al.,	21	271	0.08	[0.05; 0.12]	0.4%	9.3%
Modebe AA et al.	6	250	0.02	[0.01; 0.05]	1.0%	10.6%
Mohager MO et al.,	6	288	0.02	[0.01; 0.04]	1.3%	10.9%
Salem AK et al.,	7	203	0.03	[0.01; 0.07]	0.6%	10.0%
Igwe NN et al.,	27	500	0.05	[0.04; 0.08]	0.9%	10.6%
**Common effect model**		**3999**	**0.00**	**[0.00; 0.00]**	**100.0%**	**.**
**Random effects model**			**0.04**	**[0.01; 0.06]**	**.**	**100.0%**

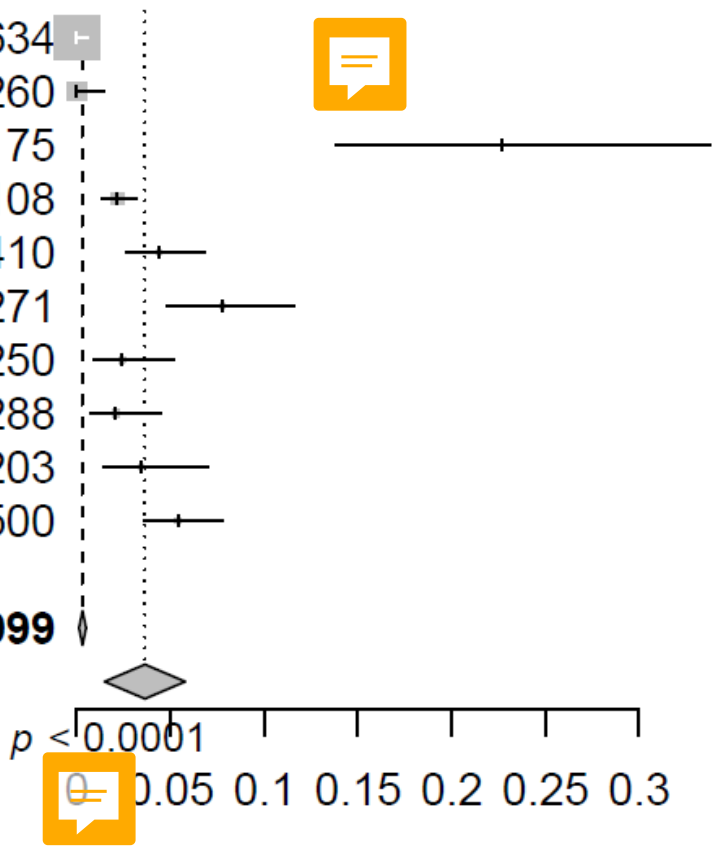

Heterogeneity: $I^2 = 92.9\%$, $\tau^2 = 0.0010$, $p < 0.0001$

Table-6: Subgroup analysis of *Schistosoma mansoni-Salmonella* co-infections in Africa

Study	Events	Total	Proportion	95%−CI	Weight (common)	Weight (random)
**Country = Ethiopia**						
Aemero M et al.,	0	634	0.00	[0.00; 0.01]	76.8%	11.6%
Beyene G et al.,	0	260	0.00	[0.00; 0.01]	13.0%	11.5%
Marege M et al.,	21	271	0.08	[0.05; 0.12]	0.4%	9.3%
**Common effect model**		**1165**	**0.00**	**[0.00; 0.00]**	**90.2%**	.
**Random effects model**			**0.02**	**[0.00; 0.07]**	.	**32.4%**
Heterogeneity: $I^2 = 91.2\%$, $\tau^2 = 0.0017$, $p < 0.0001$						
**Country = Sudan**						
Elfaki TEM et al.,	17	75	0.23	[0.14; 0.34]	0.0%	3.6%
Mohager MO et al.,	6	288	0.02	[0.01; 0.04]	1.3%	10.9%
Salem AK et al.,	7	203	0.03	[0.01; 0.07]	0.6%	10.0%
**Common effect model**		**566**	**0.03**	**[0.02; 0.04]**	**2.0%**	.
**Random effects model**			**0.09**	**[0.00; 0.21]**	.	**24.5%**
Heterogeneity: $I^2 = 88.8\%$, $\tau^2 = 0.0108$, $p = 0.0001$						
**Country = Congo**						
Kalonji LM et al.,	24	1108	0.02	[0.01; 0.03]	5.0%	11.4%
**Country = Nigeria**						
Lam PM et al.,	18	410	0.04	[0.03; 0.07]	0.9%	10.6%
Modebe AA et al.	6	250	0.02	[0.01; 0.05]	1.0%	10.6%
Igwe NN et al.,	27	500	0.05	[0.04; 0.08]	0.9%	10.6%
**Common effect model**		**1160**	**0.04**	**[0.03; 0.05]**	**2.9%**	.
**Random effects model**			**0.04**	**[0.02; 0.06]**	.	**31.8%**
Heterogeneity: $I^2 = 58.3\%$, $\tau^2 = 0.0001$, $p = 0.0908$						
**Common effect model**		**3999**	**0.00**	**[0.00; 0.00]**	**100.0%**	.
**Random effects model**			**0.04**	**[0.01; 0.06]**	.	**100.0%**

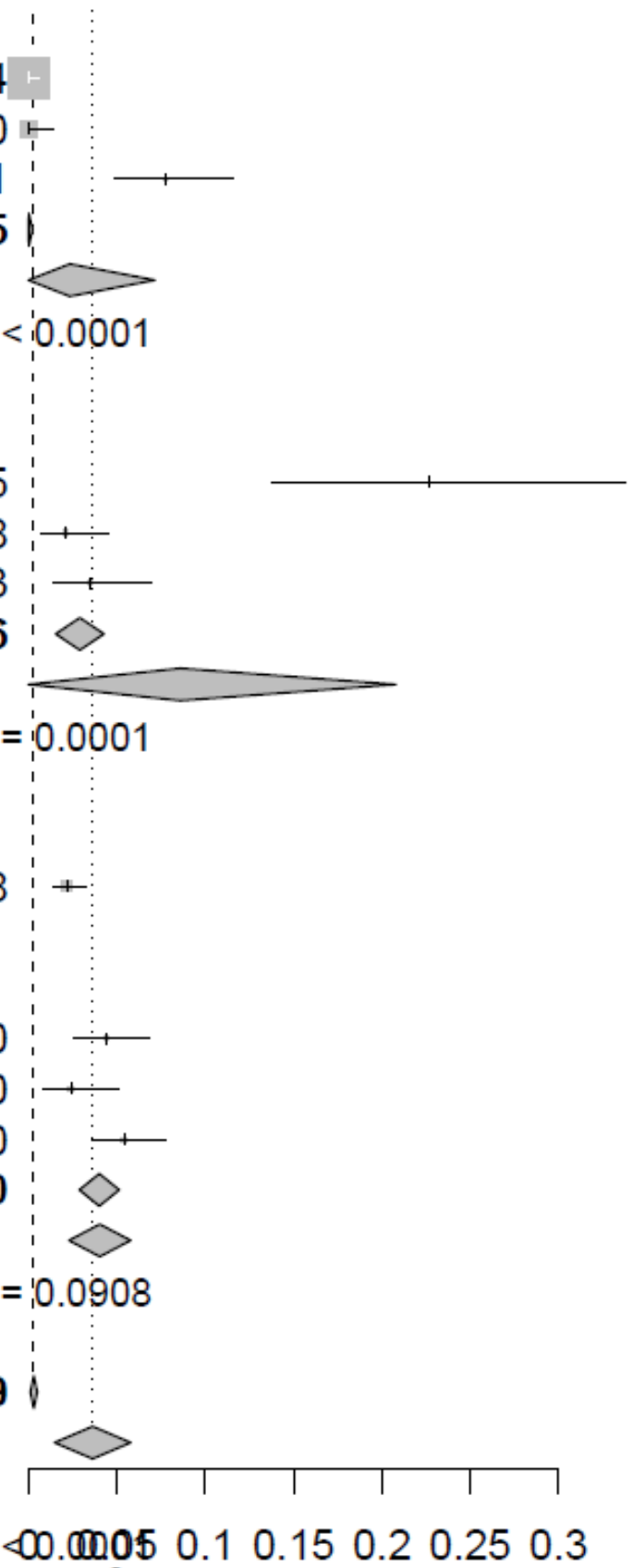

Heterogeneity: $I^2 = 92.9\%$, $\tau^2 = 0.0010$, $p < 0.0001$
Test for subgroup differences (common effect): $\chi^2_3 = 80.86$, df = 3 ($p < 0.0001$)
Test for subgroup differences (random effects): $\chi^2_3 = 4.54$, df = 3 ($p = 0.2085$)

Prevalence of *Schistosoma hematobium-Salmonella* co-infections in Africa

Among the 3170 study participants from 9 articles, the pooled prevalence of *Schistosoma hematobium-Salmonella* co-infections was 12% [0-23%] at the 95%-CI according to the random effects model **(Table-7).** Subgroup analysis by country was also performed, with a prevalence of 0% in Ethiopia, 25% in Sudan, and 8% in Nigeria (**Table-8**).

Table-7: Prevalence of *Schistosoma hematobium-Salmonella* co-infections in Africa

Study	Events	Total	Proportion	95%−CI	Weight (common)	Weight (random)
Abubakar IM et al.,	4	410	0.01	[0.00; 0.02]	2.8%	11.3%
Aemero M et al.,	0	634	0.00	[0.00; 0.01]	52.6%	11.3%
Beyene G et al.,	0	260	0.00	[0.00; 0.01]	8.9%	11.3%
Henry GB et al.,	0	505	0.00	[0.00; 0.01]	33.4%	11.3%
Lam PM et al.,	6	410	0.01	[0.01; 0.03]	1.9%	11.3%
Mohager MO et al.,	36	288	0.12	[0.09; 0.17]	0.2%	11.1%
Nury WM et al.,	52	100	0.52	[0.42; 0.62]	0.0%	10.4%
Salem AK et al.,	23	203	0.11	[0.07; 0.17]	0.1%	11.1%
Adetayo.O et al.,	108	360	0.30	[0.25; 0.35]	0.1%	11.1%
**Common effect model**		**3170**	**0.00**	**[0.00; 0.00]**	**100.0%**	**.**
**Random effects model**			**0.12**	**[0.00; 0.23]**	**.**	**100.0%**

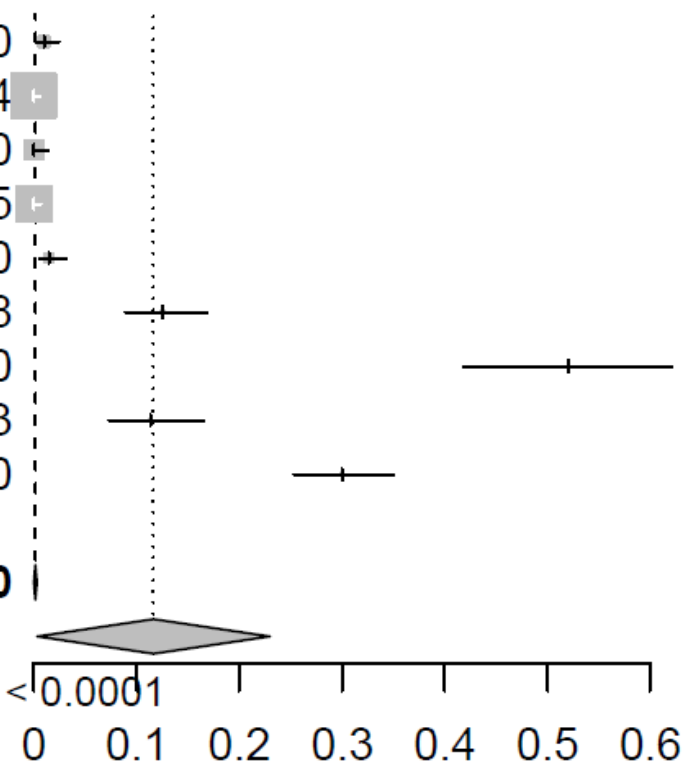

Heterogeneity: $I^2 = 97.6\%$, $\tau^2 = 0.0296$, $p < 0.0001$

Table-8: Subgroup analysis of *Schistosoma hematobium-Salmonella* co-infections in Africa

Study	Events	Total	Proportion	95%−CI	Weight (common)	Weight (random)
**Country = Nigeria**						
Abubakar IM et al.,	4	410	0.01	[0.00; 0.02]	2.8%	11.3%
Henry GB et al.,	0	505	0.00	[0.00; 0.01]	33.4%	11.3%
Lam PM et al.,	6	410	0.01	[0.01; 0.03]	1.9%	11.3%
Adetayo.O et al.,	108	360	0.30	[0.25; 0.35]	0.1%	11.1%
**Common effect model**		**1685**	**0.00**	**[0.00; 0.00]**	**38.2%**	**.**
**Random effects model**			**0.08**	**[0.00; 0.22]**	**.**	**44.8%**
Heterogeneity: $I^2 = 98.1\%$, $\tau^2 = 0.0206$, $p < 0.0001$						
**Country = Ethiopia**						
Aemero M et al.,	0	634	0.00	[0.00; 0.01]	52.6%	11.3%
Beyene G et al.,	0	260	0.00	[0.00; 0.01]	8.9%	11.3%
**Common effect model**		**894**	**0.00**	**[0.00; 0.00]**	**61.5%**	**.**
**Random effects model**			**0.00**	**[0.00; 0.00]**	**.**	**22.5%**
Heterogeneity: $I^2 = 0\%$, $\tau^2 = 0$, $p = 1.0000$						
**Country = Sudan**						
Mohager MO et al.,	36	288	0.12	[0.09; 0.17]	0.2%	11.1%
Nury WM et al.,	52	100	0.52	[0.42; 0.62]	0.0%	10.4%
Salem AK et al.,	23	203	0.11	[0.07; 0.17]	0.1%	11.1%
**Common effect model**		**591**	**0.15**	**[0.12; 0.18]**	**0.3%**	**.**
**Random effects model**			**0.25**	**[0.00; 0.51]**	**.**	**32.6%**
Heterogeneity: $I^2 = 96.6\%$, $\tau^2 = 0.0511$, $p < 0.0001$						
**Common effect model**		**3170**	**0.00**	**[0.00; 0.00]**	**100.0%**	**.**
**Random effects model**			**0.12**	**[0.00; 0.23]**	**.**	**100.0%**

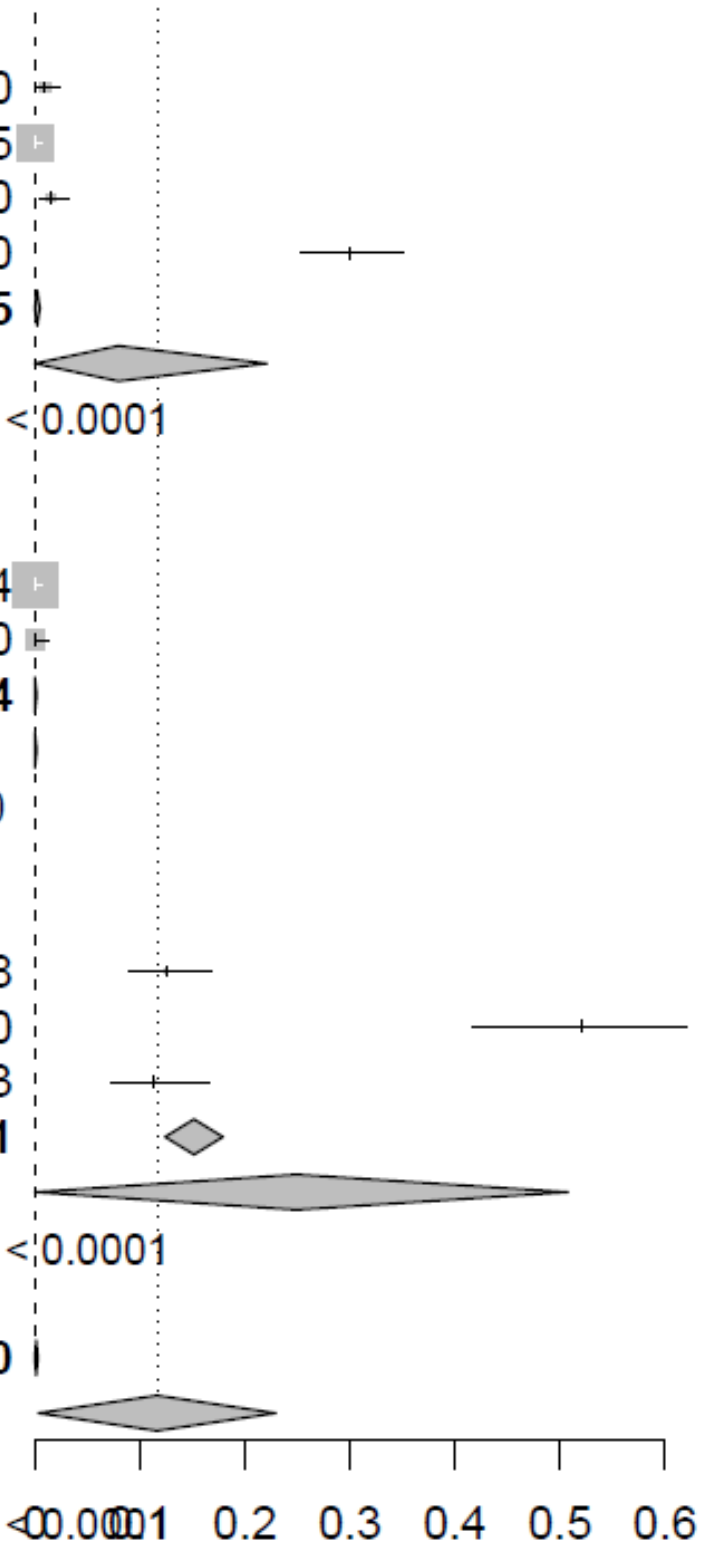

Heterogeneity: $I^2 = 97.6\%$, $\tau^2 = 0.0296$, $p < 0.0001$
Test for subgroup differences (common effect): $\chi^2_2 = 116.40$, df = 2 ($p < 0.0001$)
Test for subgroup differences (random effects): $\chi^2_2 = 4.79$, df = 2 ($p = 0.0910$)

3.2. Overview of the narrative review

Biological mechanisms of *Schistosoma-Salmonella* Co-infection

Schistosoma-Salmonella co-infection results from interconnected mechanisms involving immune modulation, tissue pathology, and direct pathogen-pathogen interactions, leading to persistent and often treatment-refractory infection. Both infections can impair hepatic and renal function, potentially altering host metabolic processes and the pharmacokinetics and pharmacodynamics of therapeutic agents. In chronic schistosomiasis, eggs become trapped in tissues, particularly the liver and other organs, where they release soluble egg antigens and proteolytic enzymes that induce persistent granulomatous inflammation. Over time, this result in fibrosis and structural damage, creating a microenvironment that facilitates secondary bacterial infections, including Salmonella (1).

The central mechanism is schistosome-driven immune modulation. Schistosome infection induces a dominant Th2 immune response characterized by increased IL-4 and IL-13 production, IgE elevation, and eosinophilia, while suppressing pro-inflammatory and Th1/Th17 cytokines. This shift promotes alternatively activated (M2) macrophages and reduces effective intracellular killing mechanisms required for Salmonella clearance (28). Further amplification of this response occurs through schistosome egg-derived immunomodulatory molecules such as IPSE/α-1, which stimulate basophils to release IL-4 and IL-13, reinforcing Th2 polarization and suppressing Th1/Th17-mediated antibacterial immunity (3, 22, 29). Additionally, expansion of regulatory T and B cells further contributes to immune suppression and bacterial persistence (29).

Direct physical interaction between the pathogens also plays a key role. *Salmonella* can colonize the tegument and intestinal tract of adult Schistosomes via fimbrial adhesins (FimH) binding to mannose receptor-like glycol-proteins on the worm surface. This interaction creates a protected intravascular niche that supports bacterial persistence and replication while reducing immune and antibiotic accessibility (20, 22, 29). Experimental studies confirm increased bacterial burden and impaired macrophage

bactericidal activity in schistosome-infected hosts, further enhancing systemic dissemination of *Salmonella* (3, 20). Clinically, co-infection is associated with chronic septicemic salmonellosis characterized by persistent bacteremia, hepatosplenomegaly, and recurrent febrile illness (17, 22, 29). Treatment failure is common when *Salmonella* infection is managed without addressing underlying schistosomiasis, as bacteria protected within or attached to worms may persist despite antibiotic therapy (21, 22).

Although most evidence indicates that schistosomiasis promotes *Salmonella* persistence through immune modulation and physical shielding, experimental studies also demonstrated a bidirectional interaction. *Salmonella typhimurium* infection has been shown to reduce *Schistosoma japonicum* worm and egg burdens, ameliorate disease pathology, and partially restore metabolic disturbances through inverse immune polarization (30). These findings suggest that the interaction between *Schistosoma* and *Salmonella* is bidirectional and highly dependent on the host immune response, parasite species, bacterial serovar, and stage of infection.

Experimental evidence further indicates that these interactions are dynamic over time and depend on the stage of schistosomiasis. During acute schistosomiasis, *Salmonella typhimurium* is associated with increased fecal shedding and reduced hepatic and splenic colonization, as well as decreased schistosome egg deposition. In contrast, chronic infection favors bacterial retention within hepatic and splenic tissues with reduced stool excretion, supporting persistent intravascular infection. Co-infection also modulates schistosomiasis-associated pathology by enhancing splenic inflammation and apoptosis while unexpectedly reducing collagen deposition and fibrosis (31).

Antimicrobial resistance and treatment failure in co-infection

A major clinical consequence of *Schistosoma*-*Salmonella* interaction is reduced antibiotic efficacy and persistent infection. *Salmonella* attached to Schistosomes via fimbrial adhesins may be physically shielded from multiple antibiotic classes, thereby reducing drug susceptibility and enhancing bacterial persistence (20, 21, 29). In this protected niche, bacteria may be exposed to sub-therapeutic antibiotic concentrations during treatment, creating selective pressure that may favor the emergence of a resistant population (21, 29). Therapeutic failure is further exacerbated by Schistosome induced immune suppression, which impairs bacterial clearance even in the presence of effective antibiotic therapy. In addition, anti-Schistosoma treatment may release previously protected bacteria into circulation following worm death, potentially contributing to recurrent bacteremia if not combined with appropriate antibacterial therapy (22, 29). Clinically nonspecific febrile presentations and overlapping symptoms contribute to delayed or misdiagnosis, increasing inappropriate antibiotic exposure and further complicating treatment outcomes (22).

Experimental antibiotic protection assays have demonstrated that *Schistosoma*-associated *Salmonella* exhibit a 4-16- fold reduction in susceptibility to eight commonly used antibiotics, mediated by FimH-dependent binding of the bacteria to the *Schistosoma* tegument (32).

4. Discussion

Schistosoma-Salmonella co-infection is a significant public health problem and a major challenge to clinical management in endemic areas (1). A recent study in co-infected patients suggests a symbiotic relationship between the bacteria and the parasite (20). This co-infection occurs when invasive *Salmonella* adheres to adult *Schistosoma*, resulting in persistent infection and various clinical manifestations (5). Untreated schistosomiasis may lead to recurrent bacteremia following *Salmonella* therapy (33). In addition, latent bacteria within *Schistosoma* reduce antibiotic effectiveness and may contribute to drug resistance (34). Co-infection is common in Africa (22), particularly among schoolchildren (35), yet its prevalence and distribution across endemic regions remain insufficiently studied.

This systematic review synthesized the prevalence of *Schistosoma-Salmonella* co-infections, *Schistosoma mansoni-Salmonella* co-infections, *Schistosoma hematobium-Salmonella* co-infections, biological mechanisms and antimicrobial resistance of the co-infections in Africa. This study highlights a concise report on co-infection, and it benefits clinicians, epidemiologists and policymakers for evidence-based management. It will also be important for local and regional health policymakers to design appropriate methods of prevention and control in Africa. To date, a co-infection report in Africa is limited and this is the first systematic review study that addresses the epidemiology, biological mechanisms, and antimicrobial resistance of *Schistosoma-Salmonella* co-infections in Africa.

This updated systematic review on recent observational data of all the available literature on the prevalence of *Schistosoma-Salmonella, Schistosoma mansoni-Salmonella* and *Schistosoma hematobium-Salmonella* co-infection included 16 records in Africa **(S-1).** Among these records, 7 eligible articles are on *Schistosoma Salmonella* co-infection with pooled prevalence was 9%, **95%-CI** [2-15%] **(Table-4).** This figure is lower than studies conducted in Sudan (76%)(36), in Nigeria (16.7%) (13), and Sudan (22.2%, 14.7%) (6, 17), and higher than the studies conducted in Nigeria (5.8%, 4.4%) (18, 31) and Ethiopia (0%,0%) (14, 15). This variation could be due to sample size variation, diagnostic methods, study populations, and sampling season.

Besides, of the 16 records, 10 articles are on *Schistosoma mansoni-Salmonella* co-infection with pooled prevalence was 4%, 95-CI [1-6%] **(Table-5).** This figure is comparable with studies reported from Nigeria (4.4%, 5.4%) (23, 31) and Sudan (3.4%) (6) and lower than reports from Egypt (9%)(37), Ethiopia (7.7%) (21) and Sudan (22.7%) (19), and higher than studies conducted in Ethiopia (0%, 0%) (14, 15), Congo (2.2%) (20), Nigeria (2.4%) (22) and Sudan (2.1%) (17). This might be due to differences in the endemicity and transmission intensity of *Schistosoma mansoni* across study areas, variations in exposure to contaminated freshwater, sanitation conditions, and infection intensity may contribute to the observed heterogeneity (2, 38, 39).

Similarly, subgroup analysis of the prevalence of *Schistosoma mansoni-Salmonella* co-infection was performed by country as follows: 2% in Ethiopia, 9% in Sudan, 4% in Nigeria and 2% in Congo **(Table-6)**. This observed country level heterogeneity may be due to the differences in *Schistosoma mansoni* endemicity and transmission intensity and implementation of schistosomiasis control programs across Ethiopia, Sudan, Nigeria, and Congo (2, 38).

Moreover, out of 16 records, 9 eligible primary articles were on *Schistosoma hematobium-Salmonella* co-infection with an overall prevalence was 12% [0-23%] (**Table-7**). This is comparable with report from Sudan (12.5%) (17) and lower than studies conducted in Nigeria (30%) (27) and Sudan (52%)(26) but higher than studies conducted in Ethiopia (0%,0%) (14, 15), Nigeria (0%) (25), Nigeria (1.46%) (31) and Sudan (11.3%) (6). This might be due to variation in control interventions and the endemicity and transmission intensity of urinary Schistosomiasis in different settings (38, 40). Similarly, subgroup analysis on the prevalence of *Schistosoma hematobium-Salmonella* co-infection was performed by country as follows: 0% in Ethiopia, 25% in Sudan, and 8% in Nigeria **(Table-8)**. This country level heterogeneity might be due to differences in the implementation control programs, chronic urinary tract pathology caused by *Schistosoma haematobium* infection and endemicity and transmission intensity of urinary schistosomiasis across Ethiopia, Sudan, and Nigeria (38, 40).

Evidences synthesized in this narrative review indicate that *Schistosoma-Salmonella* co-infection is driven by immune modulation, tissue pathology, and direct pathogen interactions, which promotes bacterial persistence. These mechanisms have important clinical implications, as they provide a biological basis for chronic bacteremia, and the need to consider both infections during diagnosis and management in endemic areas.

However, the current evidence is derived predominantly from experimental and mechanistic studies, with limited confirmation from well-designed human studies. Future research should prioritize prospective clinical and translational studies to clarify the underlying mechanisms and evaluate their implications for diagnosis, treatment, and control of *Schistosoma Salmonella* co-infection.

The available evidence suggests that treatment failure in *Schistosoma-Salmonella* co-infection is driven primarily by bacterial persistence rather than established antimicrobial resistance alone. This distinction has important therapeutic implications, as successful management require simultaneous treatment of both schistosomiasis and salmonellosis. Given that current evidence is largely experimental, strong clinical studies are needed to define the contribution of these mechanisms to antibiotic failure and antimicrobial resistance in endemic populations.

5. **Conclusions**

This review highlights *Schistosoma-Salmonella* co-infection as an overlooked public health concern in Africa, The available evidence indicates that this neglected co-infection is characterized by a considerable epidemiological burden, complex biological interactions, and important therapeutic challenges. Strengthened surveillance, early diagnosis, integrated clinical management, and further research are essential to improve control and patient outcome in endemic settings.

Limitations of the study

Only studies published in English languages and studies retrieved from open access sources were used in the analysis, which may introduce languages bias and have led to the exclusion of relevant subscription-based articles. Furthermore, due to the limited number of studies addressing the biological mechanism and antimicrobial resistance of *Schistosoma Salmonella* co-infections was not fully achieved quantitatively.

List of abbreviations and acronyms

CDC: Center of Disease Control, CI-Confident Interval, CINAHL: Cumulative Index to Nursing and Allied Health Literature, df: Degree of freedom: Fimh: Fimbrial adhesins, FMOH: Federal Ministry of Health, IgE: Immunoglobulin- E, IL: Interleukin, IPSE/α-1: Interleukin Principle Schistosoma Egg alpha-1 isomer, MeSH: Medical Science Heading, Th: T-helper cell, PRISMA-P: Preferred Reporting Items for Systemic review and Meta-Analysis Protocols, S-1: Supplementary file one- PRISMA-P diagram, S-2: Supplementary file two- PRISMA checklist, S-3: Supplementary file three-Quality appraisal JBI checklist for *Schistosoma-Salmonella* co-infection, S-4: Supplementary file four-list of tables from 1-3, SE: Standard error, WHO: World Health Organization.

Availability of data and materials

The raw data used for this study will be made available upon request

Author Contributions

Brhane Berhe Aregawi (BBA) and Abel Deres (AD) participated in conceptualization, drafting, and revision of the manuscript. Hadush Negash (HN), GB (Getachew Belay), Merhawi Alemu (MA), Aberham Abere (AA) and Tesfay Abadi (TA) participated in revision of the manuscript. Haftom Legese (HL) participated in data extraction, analysis and revision of the paper. All the authors read and approved the final manuscript.

Funding

Not applicable

Competing interests

The authors declare that they have no competing interests.

Ethics approval and consent to participate

Not applicable

Consent for publication

Individual data such as images and videos did not accompany this particular manuscript; hence, consent for publication is not applicable.

References

1.Hsiao A, Toy T, Seo HJ, Marks F. Interaction between Salmonella and schistosomiasis: a review. PLoS pathogens. 2016;12(12):e1005928.
2. Grimes JE, Croll D, Harrison WE, Utzinger J, Freeman MC, Templeton MR. The roles of water, sanitation and hygiene in reducing schistosomiasis: a review. Parasites & vectors. 2015;8(1):156.
3. Perera DJ, Koger-Pease C, Paulini K, Daoudi M, Ndao M. Beyond schistosomiasis: unraveling co-infections and altered immunity. Clinical microbiology reviews. 2024;37(1):e00098-23.
4. Organization WH. Antimicrobial resistance: fact sheet. Geneva: WHO. 2023.
5. Muniz-Junqueira MI, Tosta CE, Prata A. Schistosoma-associated chronic septicemic salmonellosis: evolution of knowledge and immunopathogenic mechanisms. Revista da Sociedade Brasileira de Medicina Tropical. 2009;42(4).
6. Salem AK, Bilal NE, Ibrahim ME. Frequent carriage of invasive Salmonellae amongst patients infected with schistosomiasis in Sudan. African Journal of Microbiology Research. 2015;9(8):543-8.
7. Maria C. Africa Population: Total Population, Growth & Country Rankings. The Fact Institute. 2026.
8. Moher D, Shamseer L, Clarke M, Ghersi D, Liberati A, Petticrew M, et al. Preferred reporting items for systematic review and meta-analysis protocols (PRISMA-P) 2015 statement. Systematic reviews. 2015;4(1):1.
9. Stroup DF, Berlin JA, Morton SC, Olkin I, Williamson GD, Rennie D, et al. Meta-analysis of observational studies in epidemiology: a proposal for reporting. Jama. 2000;283(15):2008-12.
10. Institute JB. Joanna Briggs Institute Reviewers' Manual: 2017 edition. Australia: The Joanna Briggs Institute; 2017. Cited Here. 2019.
11. Chandler J, Cumpston M, Li T, Page MJ, Welch V. Cochrane handbook for systematic reviews of interventions. Hoboken: Wiley. 2019;4(1002):14651858.
12. Egger M, Smith GD, Schneider M, Minder C. Bias in meta-analysis detected by a simple, graphical test. bmj. 1997;315(7109):629-34.
13. Obi A, Yesufu H, Fagbenro-Beyioku A, Obi C. Concurrent infections with schistosomiasis, typhoid fever and malaria among patients in Edo State, Nigeria. 1996.
14. Aemero M, Erko B, Ashenafi M, Erku W. Assessment on the Status of Salmonella and Schistosoma Coinfections in the Middle Awash Valley and Ziway, Ethiopia". Ethiopian Journal of Health and Biomedical Sciences. 2014;6(1):59-63.
15. Beyene G, Tasew H. Prevalence of intestinal parasite, Shigella and Salmonella species among diarrheal children in Jimma health center, Jimma southwest Ethiopia: a cross sectional study. Annals of clinical microbiology and antimicrobials. 2014;13(1):10.
16. Lar P, Emojevwe M, Onah J. Incidence of mixed infections of Schistosoma and Salmonella in the Federal Capital Territory, Abuja. 2006.

17. Mohager MO, Mohager SO, Kaddam LA. The association between shistosomiasis and enteric fever in a single Schistosoma endemic area in Sudan. International Journal of Pharmaceutical Sciences and Research. 2014;5(6):2181.
18. Yandev D, Chigor V, Eze E. Studies on Biochemical Characterization of Salmonella and Mixed Infection Cases with Schistosomiasis in Kwande LGA, Benue State, Nigeria.
19. Elfaki TEM, Abdalla EJ. Association between intestinal schistosomiasis and enteric fever in New Halfa City, Sudan. Int J Novel Res Healthc Nurs. 2015;2:45-52.
20. Mbuyi-Kalonji L, Barbé B, Nkoji G, Madinga J, Roucher C, Linsuke S, et al. Non-typhoidal Salmonella intestinal carriage in a Schistosoma mansoni endemic community in a rural area of the Democratic Republic of Congo. PLoS neglected tropical diseases. 2020;14(2):e0007875.
21. Marege A, Seid M, Boke B, Thomas S, Arage M, Mouze N, et al. Prevalence of Schistosoma mansoni–Salmonella coinfection among patients in southern Ethiopia. New microbes and new infections. 2021;40:100842.
22. Modebe A, Nnachi A, Ukaegbu C, Tata N, Agah M, Udu-Ibiam O, et al. Dual infections of Enteric Salmonella species with Schistosoma mansoni among Patients from two Hospitals in Jos, Nigeria. Journal of Applied and Environmental Microbiology. 2014;2(4):198-202.
23. Igwe N, Agbo E. Incidence of co-infection of enteric Salmonella with Schistosoma in Kachia Local Government Area of Kaduna State, Nigeria. International Journal of Tropical Medicine and Public Health. 2014;3(1):12-7.
24. Abubakar IM, Madaki AB, Ari JL, Kazzayo AM, Saleh AA, Iliyasu HN, et al. Co-infection of Schistosoma Haematobium and Salmonella Species Among Febrile Patients In Doma Local Government Area, Nasarawa State, Nigeria. International Journal of Medical Science and Public Health. 2025;11(2):28-.
25. HENRY GB. PREVALENCE OF URINARY SCHISTOSOMIASIS AND ITS CO-INFECTION WITH SALMONELLA SPECIES AMONG PUPILS IN JABA LOCAL GOVERNMENT AREA, KADUNA STATE, NIGERIA 2016.
26. Nury WM. Frequency of Schistosoma haematobium and Salmonella Co-infection in Khartoum-state, Sudan: Al-Neelain University; 2022.
27. ADETAYO O, DIKWA K, HAROUN A. Endemicity of Urinary Schsitosomiasis and Co-Infection with Enteric Fever Bacteria among Selected Primary Schools' Pupil in Chikun Local Government Area, Kaduna State, Nigeria. Journal of Applied Sciences & Environmental Management. 2025;29(11).
28. Schramm G, Suwandi A, Galeev A, Sharma S, Braun J, Claes A-K, et al. Schistosome eggs impair protective Th1/Th17 immune responses against Salmonella infection. Frontiers in immunology. 2018;9:2614.
29. Bajinka O, Qi M, Barrow A, Touray AO, Yang L, Tan Y. Pathogenicity of Salmonella during Schistosoma-Salmonella Co-infections and the importance of the gut microbiota. Current microbiology. 2022;79(1):26.
30. Zhu X, Chen L, Wu J, Tang H, Wang Y. Salmonella typhimurium infection reduces Schistosoma japonicum worm burden in mice. Scientific Reports. 2017;7(1):1349.
31. Lam HYP, Wu W-J, Liang T-R, Li H-C, Chang K-C, Peng S-Y. Salmonella typhimurium exacerbates injuries but resolves fibrosis in liver and spleen during Schistosoma mansoni infection. Journal of Microbiology, Immunology and Infection. 2023;56(3):477-89.

32. Barnhill AE, Novozhilova E, Day TA, Carlson SA. Schistosoma-associated Salmonella resist antibiotics via specific fimbrial attachments to the flatworm. Parasites & vectors. 2011;4(1):123.
33. Gendrel D, Richard-Lenoble D, Kombila M, Engohan E, Nardou M, Moussavou A, et al. Schistosoma intercalatum and relapses of Salmonella infection in children. The American journal of tropical medicine and hygiene. 1984;33(6):1166-9.
34. Mahmoud AA. Schistosomiasis (bilharziasis): from antiquity to the present. Infectious Disease Clinics. 2004;18(2):207-18.
35. Stothard JR, Gabrielli A-F. Schistosomiasis in African infants and preschool children: to treat or not to treat? Trends in parasitology. 2007;23(3):83-6.
36. Salih S, Subaa H, Asha H, Satir A. Salmonellosis complicating schistosomiasis in the Sudan. The Journal of Tropical Medicine and Hygiene. 1977;80(1):14-8.
37. El-Shazly A, Soliman M, El-Kalla M, Rezk H, El-Nemr H, Handousa A, et al. Studies on patients with Schistosomiasis mansoni, HCV and/or typhoid fever. Journal of the Egyptian Society of Parasitology. 2001;31(2):583-92.
38. WHO. Schistosomiasis fact sheet. 23 February, 2026.
39. Lundy SK, Lukacs NW. Chronic schistosome infection leads to modulation of granuloma formation and systemic immune suppression. Frontiers in immunology. 2013;4:39.
40. Colley DG, Bustinduy AL, Secor WE, King CH. Human schistosomiasis. The Lancet. 2014;383(9936):2253-64.

Click here to access/download
Supporting Information
Sumpplementary file (S-1)-PRISMA-2020-flow-diagram.docx

Click here to access/download
Supporting Information
Supplementary file (S-3)-JBI Checklist.docx

Click here to access/download
Supporting Information
Supplementary file (S-4)-Table 1-3.docx

Click here to access/download
Supporting Information
Supplementary file (S-2)-PRISMA_2020_checklist.docx
