# Supplementary material for: Schistosoma-Salmonella co-infection in Africa: Epidemiology, biological mechanisms, and antimicrobial resistance: A systematic review and meta analysis
Source: PLoS One. 2026 Oct 29;21(10):e0359975. doi: 10.1371/journal.pone.0359975 (PMC13718923; doi:10.1371/journal.pone.0359975)
Supplement: Supplementary file 3 [file pone.0359975.s002.docx]

| JBI Checklist, 2017 (cross-sectional studies) | Obi AA et al., | Abubakar IM et al., | Aemero M et al., | Beyene G et al., | Elfaki TEM et al., | Henry GB et al., | Kalonji LM et al., | Lam PM et al., | Marege M et al., | Modebe AA et al. | Mohager MO et al., |
| --- | --- | --- | --- | --- | --- | --- | --- | --- | --- | --- | --- |
| 1. Were the criteria for inclusion in the sample clearly defined? | Yes | Yes | Yes | No | Yes | Yes | Yes | Yes | Yes | Yes | Yes |
| 2. Were the study subjects and the setting described in detail? | Yes | Yes | Yes | Yes | Yes | Yes | Yes | Yes | Yes | Yes | Yes |
| 3. Was the exposure measured in a valid and reliable way? | Yes | Yes | Yes | Yes | Yes | Yes | Yes | Yes | Yes | Yes | Yes |
| 4. Were objective, standard criteria used for measurement of the condition? | Yes | Yes | Yes | Yes | Yes | Yes | Yes | Yes | Yes | Yes | Yes |
| 5. Were confounding factors identified? | Yes | Yes | Yes | Yes | Yes | Yes | Yes | Yes | Yes | Yes | Yes |
| 6. Were strategies to deal with confounding factors stated? | Yes | Yes | Yes | Yes | Yes | Yes | Yes | Yes | Not | Yes | Yes |
| 7. Were the outcomes measured in a valid and reliable way? | Yes | Yes | Yes | Yes | Yes | Yes | Yes | Yes | Yes | Yes | Yes |
| 8. Was appropriate statistical analysis used? | Yes | Yes | Yes | Yes | Yes | Yes | Yes | Yes | Yes | Yes | Yes |
| Overall quality appraisal | 8/8 (100%) | 8/8 (100%) | 8/8 (100%) | 8/8 (100%) | 8/8 (100%) | 8/8 (100%) | 8/8 (100%) | 8/8 (100%) | 8/8 (100%) | 8/8(100%) | 8/8(100%) |
| Including reason for exclusion | Included | Included | Included | Included | Included | Included | Included | Included | Included | Included | Included |

| JBI Checklist, 2017 (cross-sectional studies) | Nury WM et al., | Salem AK et al., | Yandev et al., | Adetayo.O et al., | Igwe NN et al., |
| --- | --- | --- | --- | --- | --- |
| 1. Were the criteria for inclusion in the sample clearly defined? | Yes | Yes | Yes | Yes | Yes |
| 2. Were the study subjects and the setting described in detail? | Yes | Yes | Yes | Yes | Yes |
| 3. Was the exposure measured in a valid and reliable way? | Yes | Yes | Yes | Yes | Yes |
| 4. Were objective, standard criteria used for measurement of the condition? | Yes | Yes | Yes | Yes | Yes |
| 5. Were confounding factors identified? | Yes | Yes | Yes | Yes | Yes |
| 6. Were strategies to deal with confounding factors stated? | Yes | Yes | Yes | Yes | Yes |
| 7. Were the outcomes measured in a valid and reliable way? | Yes | Yes | Yes | Yes | Yes |
| 8. Was appropriate statistical analysis used? | Yes | Yes | Yes | Yes | Yes |
| Overall appraisal: | 8/8 (100%) | 8/8 (100%) | 8/8 (100%) | 8/8 (100%) | 8/8 (100%) |
| Including reason for exclusion | Included | Included | Included | Included | Included |
