# Supplementary material for: Schistosoma-Salmonella co-infection in Africa: Epidemiology, biological mechanisms, and antimicrobial resistance: A systematic review and meta analysis
Source: PLoS One. 2026 Oct 29;21(10):e0359975. doi: 10.1371/journal.pone.0359975 (PMC13718923; doi:10.1371/journal.pone.0359975)
Supplement: Supplementary file 4 [file pone.0359975.s005.docx]

Brhane Berhe Aregawi, Assistant Prof, MSc, BSc

Adigrat University, College of Medicine and Health Sciences,

Department of Medical Laboratory Sciences,

Adigrat, Ethiopia, P.O. Box: 50

[birhaneberhe54@gmail.com](mailto:birhaneberhe54@gmail.com)

Dear editor

Journal of PLOS One

Thank you so much for your helpful assessment and comments on our review manuscript entitled “**Schistosoma-Salmonella co-infection in Africa: Epidemiology, Biological mechanisms, and Antimicrobial resistance. A systematic review and Meta analysis.”**

We thankful for your great effort, commitments and spend your time to review the manuscript review. We also appreciated the reviewers for their interest in our manuscript, and for all of their helpful assessments and comments. In addition, we believe that all of your advice and comments is mandatory for our manuscript scientific quality. We are attempted to understand your requested comments and general instructions for the manuscript. So we have revised the paper based on your comments and advice.

Please find below the point by point responses and the general corrections for the manuscript.

Sincerely,

Brhane Berhe Aregawi

Assistant Professor, MSc, BSc

**Point by point response for editors**

**Requested comment 1:** Please ensure that your manuscript meets PLOS ONE's style requirements, including those for file naming. The PLOS ONE style templates can be found at 
<https://journals.plos.org/plosone/s/file?id=wjVg/PLOSOne_formatting_sample_main_body.pdf>

and <https://journals.plos.org/plosone/s/file?id=ba62/PLOSOne_formatting_sample_title_authors_affiliations.pdf>

**Response: Dear editor, we have checked and reviewed**

**Requested comment 2:** In the online submission form, you indicated that the raw data used for this study will be made available upon request. All PLOS journals now require all data underlying the findings described in their manuscript to be freely available to other researchers, either 1. In a public repository, 2. Within the manuscript itself, or 3. Uploaded as supplementary information.
This policy applies to all data except where public deposition would breach compliance with the protocol approved by your research ethics board. If your data cannot be made publicly available for ethical or legal reasons (e.g., public availability would compromise patient privacy), please explain your reasons on resubmission and your exemption request will be escalated for approval.

**Response**: Dear editor, thank you for your valuable concern. However, all required raw data included in the manuscript as clearly indicated in **table 1** and **table 2**. Thank you for your understanding.

**Requested comment 3:** Please amend your authorship list in your manuscript file to complete the author name “Brhane Berhe”

**Response:** Dear editor, it is well acknowledged and amended it accordingly.

**Requested comment 4:** Please include captions for your Supporting Information files at the end of your manuscript, and update any in-text citations to match accordingly. Please see our Supporting Information guidelines for more information: <http://journals.plos.org/plosone/s/supporting-information>.

**Response:** Dear editor, we really thank you for this concern and included at the end of the reference of the mother document of this manuscript.

**Requested comment 5:** If the reviewer comments include a recommendation to cite specific previously published works, please review and evaluate these publications to determine whether they are relevant and should be cited. There is no requirement to cite these works unless the editor has indicated otherwise.

**Response:** Reviewed.

**Point by point response for reviewer ≠ 1**

**Requested comment 1:** Methods. While it has been stated that the PRISMA process has been followed, the methods are confused and unrepeatable, and likely not reflective of PRISMA. It is unclear how many searches were used, and where, and no inclusion or exclusion criteria are provided. This makes it impossible for future researchers to repeat the methods. If the work is to be resubmitted, there needs to be a clear set of repeatable instructions as to what was searched, and where, and what was extracted.

**Response:** Dear reviewer, I thank for your suggestion and it was clearly addressed in the methods section from page 4-6.

**Requested comment 2:** Grammar. Currently major grammar concerns are present in the work and some of these affect the legibility and meaning of sentences. The work requires a professional proof read as a result.

**Response:** Dear reviewer, the manuscript is well revised for grammar concerns.

**Requested comment 3:** Statistical analysis is unclear and p values are not clearly provided**.**

**Response:** Dear reviewer, it well addressed and revised in the mother document of the manuscript statistical data analysis section, page 8.

**Requested comment 4:** Limitations. The exclusion of non-open access work is not explained. The work should be re-analyzed with these included. Similarly, some attempt should be made to address the non-English papers, especially as Africa is the search area and many other languages are used primarily.

**Response:** Dear reviewer, thank you for this question but is it well acknowledged and stated in limitation section of this manuscript, page 24.

**Requested comment 5**: Several citations are missing in the work and the reference list requires some reformatting.

**Response:** Dear reviewer, it is well revised for citations missing in the mother document of the manuscript.

**Requested comment 6:** There is mention of a narrative review in the results; this is not introduced anywhere else in the work.

**Response:** Dear reviewer, it is well addressed in the method section, study selection and data extraction sub-section page 7, and in the eligibility criteria sub-section, page 7.

**Abstract**

**Requested comment 7:** Is there any reason why the abstract is broken?

**Response:** Dear reviewer, consider it and amend it into unstructured manner.

**Requested comment 8:** R is not important to mention. Provide more detail on the method instead.

**Response:** Dear reviewer, it is revised to “A systematic review and Meta-analysis were conducted following the PRISMA framework. The pooled prevalence of co-infection was estimated using a random effects model, with at 95% confidence intervals. Between studies heterogeneity was assessed using Cochran’s Q test and the I² statistic. Subgroup analyses were performed according to Schistosoma species and country to explore potential source of heterogeneity.”

**Requested comment 9:** Please add “-“in the abstract section conclusion sub-section

**Response:** Dear reviewer, it is well addressed.

**Requested comment 10:** Some of the keywords are already in the title, select new terms and remove duplicates

**Response:** Acknowledged and corrected to” Africa, Co-infection, Epidemiology, Incidence, Prevalence, Salmonella, Schistosoma”.

**Background and justification, page 3**

**Requested comment 11**: Please check the sentence grammar in first paragraph, line2, page 3?

**Response:** Checked and revised.

**Requested comment 12**: Please remove “a” in first paragraph, line4, page 3

**Response**: Removed

**Requested comment 13**: Please check the sentence grammar in paragraph 2, line4, page 3?

**Response:** Checked and revised

**Requested comment 14**: Please add “s” to the word “area” in paragraph 3, line6, page 3?

**Response**: Revised to “areas”

**Requested comment 15**: Please change “systemic” to the word “Systematic” in the last paragraph page 4, line 8?

**Response**: Corrected to “systematic”

**Material and Methods**

**Requested comment 16: method section, study setting subsection;** This systematic review was conducted to study the epidemiology, biological mechanisms, and antimicrobial resistance of Schistosoma-Salmonella co-infection in Africa, with a geographical area of 30.37 million km2 and an over 1.56 billion people, the second largest continent in the world next to Asia (7). Is this **in terms of people or space?**

**Response:** Dear reviewer, it well revised to**”** This systematic review was conducted to study the epidemiology, biological mechanisms, and antimicrobial resistance of Schistosoma-Salmonella co-infection in Africa. It is the world largest continent by geographic area and the second most populous continent in the world next to Asia, covering approximately 30.37 million km^2^ and hosting more than 1.56 billion people”.

**Requested comment 17: method section, protocol design sub section;** this systematic review summarized evidence exclusively observational studies (cross sectional) that were conducted on the basis of studies reported across different regions in Africa. Please check sentence grammar, this does not make sense?

**Response:** Dear reviewer, it is checked and revised to**”** This systematic review summarized evidence exclusively from observational studies with a cross-sectional study design conducted across different regions of Africa.”

**Requested comment 18: in the method section page 4, protocol design sub section line 4;** how was the PRISMA process used? The search methods do not follow these methods and are unrepeatable and….

**Response:** Dear reviewer, it is well revised to “It was performed in accordance with the Preferred Reporting Items for Systematic Reviews and Meta-analyses (PRISMA) guidelines and checklist and meta-analysis of observational studies in epidemiology guidelines. PRISMA was used to guide the transparent reporting of the identification, screening, eligibility assessment, and inclusions of the studies. The study selection process was presented using a PRISMA flow diagram, which documented the number of records identified, screened, excluded, and included in the final review. Despite the PRISMA checklist was used, a formal review protocol was not registered”.

**Requested comment 19:** the search strategy and source of the method section page 4 unclear the first few sentences contracted the later sentence and need to state exactly where was searched and what?

**Response:** Dear reviewer, thank you for your valuable comment and it revised to” A comprehensive literature search was conducted in PubMed, Google Scholar, EMBASE, Cochrane, Global Health and CINAHL. The searches were performed without date restrictions, and the final search was conducted on July 5, 2026. Only studies published in English were considered. A search strategy was developed using specific key concepts in our research questions, such as the Prevalence, Epidemiology, Biological mechanism, Antimicrobial resistance of Schistosoma Salmonella co-infection in Africa. For each key concept, appropriate free-text words and Medical Subject Headings (MeSH) were developed. The free-text words (truncated or with wildcards) and MeSH terms were combined via Boolean logic operators: “AND” and “OR”. The search strategy for each database was independently validated by three authors. We used the following search words in PubMed: (((("Prevalence"[Mesh]) OR "Epidemiology"[Mesh]) OR “Biological mechanism” AND "Schistosoma Salmonella co-infection"[Mesh]) OR "Antimicrobial resistance” [Mesh]))))). We also used keywords such as “Prevalence and Schistosoma mansoni co-infection in Africa”, “Epidemiology and Schistosoma mansoni Salmonella co-infection in Africa”, “Incidence and Schistosoma mansoni salmonella co-infection in Africa”, “Prevalence and Schistosoma haematobium Salmonella co-infection in Africa”, “Epidemiology and Schistosoma haematobium Salmonella co-infection in Africa”, and “Incidence and Urinary schistosomiasis Salmonella co-infection in Africa”.

**Requested comment 20**: Missing relevant studies and gray literature need to be explicitly stated and inclusion and exclusion criteria need to clearly stated.

**Response:** Dear reviewer, it is well revised to “To minimize the risk of missing potentially relevant studies and to identify unpublished data, supplementary searches were conducted in addition to the electronic database searches. The reference lists of all included studies and relevant systematic or narrative reviews identified during the screening process were manually screened for additional eligible studies. We also searched the official websites of relevant organizations, including the World Health Organization (WHO), the Centers for Disease Control and Prevention (CDC), and Ministry of Health (MOH), for relevant reports and other potentially eligible sources. Google Scholar was also searched using combinations of key terms related to Schistosoma, Salmonella, co-infection, and Africa. Furthermore, the Conference Proceedings Citation Index–Science (CPCI-S) was searched for relevant proceedings of the International Union Against Schistosomiasis conferences, and ProQuest Dissertations & Thesis was searched for relevant dissertations and thesis’s. These supplementary searches were performed to identify additional published, unpublished, and grey-literature sources that may not have been retrieved through the electronic database searches”.

**Requested comment 21**: In the method section, study selection and data extraction sub section, Microsoft excel version should be stated

**Response:** Dear reviewer, it is revised to “Microsoft excel 2007”.

**Requested comment 22:** Check formatting for research gate

**Response:** Dear reviewer, it is revised and corrected to: ResearchGate

**Requested comment 23**: full name of JBI checklist is needed

**Response:** Dear reviewer, checked and corrected to: Joanna Briggs Institute checklist

**Requested comment 24**: Why the author only includes open access articles?

**Response:** Dear reviewer, this is well addressed in the limitation of the study page 24 of the mother manuscript

**Requested comment 25**: Define subgroup analysis

**Response:** Dear reviewer**,** it is defined to “Subgroup analyses were conducted to explore potential sources of heterogeneity by study country. Sensitivity analyses were also performed to assess the robustness of the pooled estimates by sequentially excluding each individual study from the meta-analysis. Publication bias was not assessed because the number of included studies was fewer than the minimum of 10 studies generally recommended for reliable assessment of publication bias**”.**

**Result section**

**Requested comment 26**: Is this figure, 104 publication records with duplicates??

**Response**: Yes

**Requested comment 27**: The PRISMA flowchart need to be in the main text

**Response:** Thank you for your suggestion and well incorporated in the main text of the manuscript

**Requested comment 28:** A geographic map of prevalence would be useful for demonstrating this” Subgroup analysis by country was also performed, with a prevalence of 2% in Ethiopia, 9% in Sudan, 2% in Democratic republic of Congo, and 4% in Nigeria”

**Response:** Dear reviewer, it well acknowledge and incorporated in the mother document. **Requested comment 29: Table-5**. Tables should be condensed together and figure should be provided separately, and statistical outcomes should be reported in the main text

**Response**: Dear reviewer, I really appreciate for this comment but tables can’t be condensed because they are different things and figures are provided separately. Besides, statistical outcomes are well incorporated in the main text.

**Requested comment 30**: on the narrative review section. Has not been introduced elsewhere previously and there a lot of citation to be considered.

**Response:** Dear reviewer, I have addressed this scenario in requested comment 6. It is well addressed in the method section, study selection and data extraction sub-section page 7, and in the eligibility criteria page 7, but in regards to the citation issues, it is well acknowledged and addressed in the main text of narrative review section page 19.

**Discussion section**

**Requested comment 31**: In discussion section of the first paragraph line 1, it says do not use significance unless referring statistical significance

**Response**: Dear reviewer well accepted and revised it accordingly in the main text of the manuscript.

**Requested comment 32:** In the limitation of the study, “Furthermore, due to the limited number of studies addressing the biological mechanism and antimicrobial resistance of Schistosoma Salmonella co-infections was not fully achieved quantitatively”. This does not give sense grammatically

**Response:** Dear reviewer, it is well revised to “Moreover, due to the limited number of studies addressing the biological mechanisms and antimicrobial resistance of Schistosoma-Salmonella co-infections, these aspects could not be quantitatively synthesized and were therefore assessed narratively”.

**Requested comment 33**: The reference should be checked for consistency of formatting, page number are missing and scientific name is not italic

**Response:** Dear reviewer, Checked and revised accordingly.

**Point by point response for reviewer ≠ 2**

**Requested comment 1:** Reviewer #2: General Evaluation:
This manuscript presents a systematic review and meta-analysis evaluating the pooled prevalence, biological mechanisms, and antimicrobial resistance implications of Schistosoma Salmonella co-infections in Africa. The topic addresses a critical neglected tropical disease intersection and provides useful synthesis for clinicians and public health planners. However, several methodological and reporting refinements are required before the manuscript meets PLOS ONE’s publication standards.

**Response:** Dear reviewer, it is well considered all the raised concerns as per your suggestion.

**Requested comment 2:** Protocol Registration and Transparency:
The authors note in Section 2.2 that a formal review protocol was not registered. Given PLOS ONE’s emphasis on transparency and adherence to PRISMA guidelines, the authors must explicitly justify this omission in the text and clarify the internal safeguards implemented to minimize selection and reporting bias during screening and extraction.
**Response:** Dear reviewer**,** this systematic review was not registered in PROSPERO. The review was initiated before prospective protocol registration was undertaken. Nevertheless, predefined eligibility criteria, a structured literature-search strategy, standardized study screening procedures, and a pre-specified data-extraction framework were used to promote consistency and minimize selection and reporting bias. Study selection and data extraction were conducted systematically, with disagreements resolved through discussion and consensus. The absence of prospective protocol registration is acknowledged as a limitation of this review.

**Requested comment 3:** Addressing Extreme Statistical Heterogeneity:
The meta-analyses demonstrate extremely high heterogeneity (e.g., I^2 = 97.0% for overall co-infections; I^2 = 97.6% for S. haematobium co-infections). While random-effects models and country-level subgroup analyses were performed, residual heterogeneity remains pronounced. The Discussion section should critically expand on the underlying drivers of this variance such as differences in diagnostic modalities (microscopy vs. culture vs. PCR), participant demographics (schoolchildren vs. clinical patients), and sampling frameworks.

**Response:** Dear reviewer, thank you for this important comment. We agree that the very high statistical heterogeneity observed in the meta-analyses requires a more comprehensive interpretation. We have now discuss and incorporate differences in diagnostic modalities used to detect Schistosoma and Salmonella infections, study populations, sampling frameworks, study settings, sample sizes, and local epidemiological conditions as a possible reason for the expected heterogeneity as per your suggestion.

**Requested comment 4:** Reproducibility of the Search Strategy:
The search syntax provided in Section 2.3 contains unbalanced and redundant parentheses. The authors should provide the exact, clean search string utilized for at least one major database (e.g., PubMed) to ensure full reproducibility.

**Response**: Dear reviewer, the method section particularly the search strategy is revised and a clean search string for PubMed is also provided.

**Requested comment 5**: Distinguishing Clinical vs. Experimental Evidence:
While the narrative review commendably details biological mechanisms (Th2 immune modulation, M2 macrophage polarization, and FimH-mediated bacterial attachment), the text should more explicitly distinguish which mechanisms have been validated in human clinical studies versus those derived solely from animal/experimental models.
**Response: Dear reviewer,** thank you for this valuable comment. It is well acknowledged and addressed in the mother document narrative review section, page 24.

**Requested comment 6:** - Terminology: Correct multiple instances of "systemic review" to "systematic review".

**Response:** Dear reviewer, it is well acknowledged, revised and corrected to “systematic review” throughout the manuscript.

**Requested comment 7:** Table Formatting: Clean up minor formatting anomalies in Table 5 (e.g., stray hyphens in data alignment columns).

**Response:** Revised

**Requested comment 8:** Taxonomy: Ensure consistent italicization and capitalization for all scientific binomials (e.g., Schistosoma mansoni, Schistosoma haematobium, Salmonella typhimurium).
**Response:** Dear reviewer acknowledged and amends accordingly throughout the manuscript.
