# Supplementary material for: Schistosoma-Salmonella co-infection in Africa: Epidemiology, biological mechanisms, and antimicrobial resistance: A systematic review and meta analysis
Source: PLoS One. 2026 Oct 29;21(10):e0359975. doi: 10.1371/journal.pone.0359975 (PMC13718923; doi:10.1371/journal.pone.0359975)
Supplement: Supplementary file 5 [file pone.0359975.s006.docx]

Brhane Berhe Aregawi, Assistant Prof, MSc, BSc

Adigrat University, College of Medicine and Health Sciences,

Department of Medical Laboratory Sciences,

Adigrat, Ethiopia, P.O. Box: 50

[birhaneberhe54@gmail.com](mailto:birhaneberhe54@gmail.com)

Dear editor

Journal of PLOS One

Thank you so much for your helpful assessment and comments on our review manuscript entitled “**Schistosoma-Salmonella co-infection in Africa: Epidemiology, Biological mechanisms, and Antimicrobial resistance. A systematic review and Meta analysis.”**

We thankful for your great effort, commitments and spend your time to review the manuscript review. We also appreciated the reviewers for their interest in our manuscript, and for all of their helpful assessments and comments. In addition, we believe that all of your advice and comments is mandatory for our manuscript scientific quality. We are attempted to understand your requested comments and general instructions for the manuscript. So we have revised the paper based on your comments and advice.

Please find below the point by point responses and the general corrections for the manuscript.

Sincerely,

Brhane Berhe Aregawi

Assistant Professor, MSc, BSc

**Point by point response for editors**

**Requested comment 1:** Please review your reference list to ensure that it is complete and correct

**Response: Dear editor, we have carefully reviewed our references, checked and we have not included retracted reference articles.**

**Point by point response for reviewer ≠ 2**

**Requested comment 1:** Please verify the consistency of the PRISMA flow-diagram numbers

**Response:** Dear reviewer, I thank for your suggestion and it is clearly verified the consistency of PRISMA flow-diagram numbers and all are correctly labeled.

**Requested comment 2:** Clarify the final database search strategy and statistical-analysis details

**Response:** Dear reviewer, I thank you for your valuable comments. I have well addressed in method section search strategy subsection page 6.

**Requested comment 3:** Resolve the inconsistent statements regarding publication-bias assessment and the odds-ratio analysis

**Response:** Dear reviewer, it well addressed and revised in the mother document of the manuscript statistical data analysis section, page 8.

**Requested comment 4:** Ensure that the interpretation of antimicrobial resistance and biological mechanisms remains appropriately cautious

**Response:** Dear reviewer, thank you for this question but is it well acknowledged and well revised in the discussion section from page 23-24.

**Requested comment 5**: A final check of language, scientific nomenclature, and supplementary-file references is also recommended

**Response:** Dear reviewer, it is well considered and revised as per your suggestion.

**Thank you once again, Dear editor and reviewers**
